# An Improved Elk Herd Optimiser (IEHO)

**DOI:** 10.3390/biomimetics11080527

**Published:** 2026-07-25

**Authors:** Yanjiao Wang, Fei Du, Li Chuai

**Affiliations:** School of Electrical Engineering, Northeast Electric Power University, 169 Changchun Road, Jilin 132012, China; 20132485@neepu.edu.cn (Y.W.); 2202400397@neepu.edu.cn (L.C.)

**Keywords:** elk herd optimization algorithm, population partitioning approach, opposition-based searching, novel diversity assessment scheme, adaptive parameter tuning policy

## Abstract

The Elk Herd Optimiser (EHO) is a novel metaheuristic algorithm inspired by the reproductive behaviour of elk herds. However, it suffers from insufficient convergence accuracy and population diversity. To address these issues, this study proposes an improved EHO (IEHO). A novel individual update strategy for the breeding phase is introduced to meet the requirements of convergence speed and diversity during evolution. A new population grouping strategy is also developed to achieve a dual balance between elite guidance and spatial distribution. Cauchy distribution sampling is used to generate learning weights, and population diversity is adopted to control the step size of movement, allowing real-time monitoring and supplementation of population diversity. A differentiated learning strategy based on fitness ranking divides individuals into high-quality and ordinary categories, implementing elite guidance and swarm intelligence learning, respectively. A hybrid evolutionary mechanism, integrating reverse learning driven by generalised opposition and perturbation based on the Cauchy distribution, substantially strengthens the algorithm’s resistance to entrapment in local optima. Moreover, dimension-masked crossover operations are introduced to greatly optimise the efficiency of population information sharing. Finally, through comparative experiments to verify the overall performance of IEHO on the CEC2017 test suite, compared with other competing algorithms, IEHO achieves the highest number of optimal solutions across multiple test functions.

## 1. Introduction

Numerous engineering and scientific challenges encountered in real-world applications [1,2,3,4,5,6] can be formulated as optimisation problems featuring high dimensionality [7], strong nonlinearity [8], and multimodal solution spaces [9]. When addressing such problems, conventional mathematical programming techniques suffer from prohibitively high computational costs [10] and a marked tendency to converge to suboptimal local solutions. In contrast, metaheuristic algorithms—owing to their model-independent search mechanisms, conceptual simplicity, and straightforward implementation—have gained widespread acceptance as effective tools for solving complex optimization tasks. These algorithms simulate intelligent behaviours observed in nature or biological populations—exemplified by genetic algorithms (GAs) [11], differential evolution (DE) [12], particle swarm optimisation (PSO) [13], and simulated annealing (SA) [14]—and employ stochastic search strategies that are independent of gradient information to perform global exploration and local exploitation within the solution space. Compared with traditional methods, metaheuristic algorithms exhibit superior versatility and robustness, capable of effectively handling a wide range of complex optimisation problems characterised by nonlinearity and nondifferentiability. Consequently, they have become a focal point of research in the optimisation community. At present, research on metaheuristic algorithms primarily advances along two main directions. The first direction focuses on the improvement and integration of existing mature algorithms to enhance their convergence accuracy, speed and stability. The second direction is dedicated to the design of novel algorithms that precisely emulate underutilised intelligent behaviours observed in nature, thereby expanding the applicability of metaheuristic approaches across diverse scenarios.

In terms of algorithmic improvement, researchers have been devoted to balancing the exploration and exploitation capabilities of algorithms by introducing novel mechanisms, enhancing their optimisation performance. Representative advances in recent years include the following: Rodan et al. (2025) proposed an improved frilled lizard optimiser (EFLO) [15], which enhances population diversity through a novel escape strategy and a dynamic learning mechanism. Liu et al. (2025) introduced an improved whale optimisation algorithm (IWOA) [16] that incorporates a new population initialisation method and adaptive parameters to balance exploration and exploitation. Gu et al. (2025) developed an improved dung beetle optimiser (IDBO) [17], which integrates adaptive parameters with multiobjective strategies to effectively enhance the global search capability and convergence accuracy, hence improving overall performance. Shaikh et al. (2025) proposed an improved starfish optimisation algorithm for global optimisation problems, which employs multiple novel search equations and utilises dynamic parameter control to adaptively adjust the balance between exploitation and exploration, thus strengthening the algorithm’s overall capability [18].

In the realm of novel algorithm design, researchers have continuously drawn inspiration from natural phenomena. For instance, Ouyang et al. (2025) proposed the beaver behaviour optimiser (BBO) [19], inspired by the dam-building behaviour of beavers in nature, which simulates the social cooperation, strategic planning, and environmental adaptability demonstrated by beavers during dam construction. In 2025, M. Song et al. developed the octopus optimisation algorithm (OOA) [20] by investigating the diverse foraging strategies employed by octopi in nature. Bo Yang et al. (2025) introduced the caterpillar fungus optimise (CFO) [21], which simulates the key behaviours observed in the life cycle of Ophiocordyceps sinensis, leveraging the synergistic interplay of four strategies—mycelial infection, spore dispersal, parasitism and spiral movement—to balance global search and local convergence. Gámez (2025) proposed the Tetragonula carbonaria optimiser algorithm (TGCOA) [22] by modelling three critical behaviours of stingless bee hive construction: brood chamber construction, honey pot construction, and wax source foraging. These novel algorithms offer fresh perspectives and promising avenues for addressing complex optimisation problems.

Against this backdrop, the Elk Herd Optimiser (EHO) proposed by Mohammed et al. in 2024 [23] is a novel metaheuristic algorithm inspired by the reproductive behaviour of elk herds, characterised by its clear structure and relatively few parameters. However, an in-depth analysis of its operational mechanisms reveals several inherent deficiencies that critically constrain its optimisation performance. Firstly, during the rutting stage, the algorithm directly selects individuals with the best fitness values as stags, which tends to cause stag individuals to cluster within the solution space. If these individuals are situated near local optima, the entire population may lose opportunities to explore other promising regions. Meanwhile, the roulette-wheel-based family allocation mechanism becomes increasingly random in later evolutionary stages as fitness differences amongst individuals diminish, preventing superior stags from obtaining sufficient reproductive resources and thereby weakening the convergence directionality of the algorithm. Secondly, during the calving stage, the position update formulas for stags and hinds rely on random individuals and parameters, respectively, resulting in considerable blindness in search directions, a lack of effective utilisation of high-quality genetic information, and an absence of adaptive step-size control. These deficiencies collectively give rise to insufficient convergence accuracy and rapid loss of population diversity in the EHO algorithm.

To address the aforementioned shortcomings of the EHO algorithm, several improvement efforts have been undertaken. For instance, the enhanced EHO (EQDEHO) [24] attempts to introduce a dynamic elite mutation strategy to enhance the quality of optimal solutions. However, it pays relatively insufficient attention to the coordinated design of the overall search trajectory and population update mechanisms. In view of this gap, on the basis of an in-depth analysis of the population grouping strategy and individual update mechanisms of the EHO algorithm, this study proposes an improved EHO (IEHO). The main contributions of this study are as follows.

(1) Improved rutting stage. A dual-criterion grouping strategy of ‘elite guidance plus spatial dispersion’ is proposed to address the issues of stag aggregation and uneven family resource allocation in the original EHO algorithm. The individual with the optimal fitness in the population is selected as the initial stag. From the top 20% of high-quality individuals ranked by fitness, the remaining stags are sequentially selected by maximising the minimum Euclidean distance to the already selected stags, thus ensuring a uniform distribution of the stag population across the solution space and mitigating, to a certain extent, the rapid loss of population diversity caused by stag clustering. On this basis, each stag forms a family group with the spatially nearest individuals centred around itself, whilst individuals not covered by any family are assigned to a free individual group. This grouping mechanism not only preserves the fast convergence capability endowed by elite guidance but also effectively maintains population diversity through spatially dispersed distribution, thereby alleviating the premature convergence phenomenon to a certain degree.

(2) Enhanced calving stage. A dynamic step-size control strategy based on Cauchy distribution sampling and population diversity feedback is developed to overcome the lack of adaptive step size control and excessive randomness in evolutionary directions of the original EHO algorithm. A truncated Cauchy distribution is employed to generate learning weights, enabling the step size to adaptively adjust in accordance with changes in population diversity—when diversity declines, the step size increases appropriately to enhance exploration; otherwise, it decreases to facilitate refined exploitation. After the individual position update is completed, a dimensional mask mechanism is introduced to perform dimensional crossover operations on individuals with a certain probability. This strategy not only preserves the evolutionary achievements on converged dimensions but also continuously activates the search potential of insufficiently explored dimensions, hence achieving a synergistic balance between exploitation and exploration.

(3) Novel learning strategy. During the evolution of stag individuals, a dynamically adjusted backward learning trigger probability is introduced in accordance with the iterative process—the probability remains low in early iterations to maintain the stability of the evolutionary direction, and it is moderately increased in later iterations to enhance the ability to escape local optima. When backward learning is triggered, the algorithm generates backward solutions for the stag individuals, compares the fitness of the original solutions with that of the backward solutions, and retains the better ones. This refined backward learning mechanism effectively strengthens the algorithm’s capability to escape from local optimal regions whilst ensuring the convergence stability of stag individuals. Experimental results on the CEC2017 test suite demonstrate that compared with seven other competitive algorithms with excellent performance, the proposed IEHO algorithm exhibits significant superiority in terms of convergence accuracy, convergence speed, and algorithmic stability.

The remainder of this paper is organised as follows: Section 2 describes the basic principles and implementation procedure of the original EHO algorithm. Section 3 provides an in-depth analysis of the deficiencies in the optimisation process of the original EHO and presents a series of improvement strategies, constructing the IEHO algorithm. Section 4 offers a detailed discussion of the performance of IEHO based on the CEC2017 test suite. Section 5 summarises this work and the core contributions of the IEHO algorithm.

## 2. Elk Herd Optimiser

In nature, elk belong to the deer family and are highly social, forming families for reproduction during the rutting season. Motivated by the aforementioned conduct, Mohammed introduced the Elk Herd Optimiser (EHO) in 2024. In the EHO algorithm, individuals represent the position information of elk, while the fitness value serves as a quantitative indicator of the superiority of each elk’s position. It mainly includes the rutting phase and the calving phase, as detailed below. Algorithm 1 gives the pseudocode of EHO.
**Algorithm 1: The Pseudo-Code of EHO**input: Population size: EHS, Problem dimension: D, Lower limit of the search range: lb, Upper limit of the search range: ub, Proportion of bulls: Br, Maximum number of iterations: Max_iter, Number of individuals per family: G **output**: Optimal individual along with its corresponding fitness1.  Initialization of decision variables (EHS, D, Max_iter, ub, G, lb.)2.  Randomly generate an initial population $X_{i}$ containing EHS individuals within the domain3.  Calculate the fitness value $f(x_{i})$ for each individual $X_{i}$4.  t = 15.  for t = 1 to Max_iter do6.  //Rutting phase of Section 2.17.  Sort the EHS individuals in the population by fitness8.  Determine the set of stag individuals Male, with size c = |EHS × Br|9.  Use the method in Section 2.1 to assign each female to the corresponding stag, forming the family groups family_i corresponding to each stag $\left\{family_{1},family_{2}\ldots family_{male}\right\}$10.// Calving phase of Section 2.211. for i = 1 to |Male| do12.      family i Each elk in family_i updates its position according to the method in Section 2.2 to produce corresponding calves13.   end for14.// Selection phase15.Merge the original population and all newly generated calves, select the top EHS individuals with better fitness as the next generation population16. t = t + 117.end for18.Retrieve the final best solution together with its objective function value.

### 2.1. Rutting Phase

During the breeding season, male elk compete for mating opportunities with females, with stronger males having higher reproductive rights. The EHO algorithm simulates this rutting reproductive behaviour of elk to determine male elk and the females that mate with them, ultimately determining the families centred around each male elk. The specific method is as follows: Assume the rutting male rate is Br, generally Br = 0.2, then the number of stags in the population should be |Br×EHS|. First, select the top |Br×EHS| better individuals in the population as stags, forming the Male population, and the remaining individuals form the Female population. Then, each female individual selects a stag from the Male population according to the probability $P_{i}$ shown in Formula (1) using a roulette wheel selection mechanism, and enters its reproductive family group. Finally, |Br×EHS| reproductive families family_i are formed with each stag as the core: $\left\{family_{1},family_{2}\ldots family_{\left|male\right|}\right\}$

(1) $$P_{i}=\frac{f(Male_{i})}{\sum_{i=1}^{\left|Male\right|}f(Male_{i})}$$

where |Male| is the number of the Male population, and $f(Male_{i})$ denotes the fitness value of the ith male stag within the male subpopulation.

### 2.2. Calving Phase

After stags and females have formed families, members of each family enter the following calving phase: For the stag in the family, a new calf is generated according to the paternal inheritance pattern shown in Formula (2); for the female in the family, a new calf is generated according to the maternal reproduction pattern shown in Formula (3).

(2) $$X_{Male,i}^{t+1}(j)=X_{Male,i}^{t}(j)+\alpha \times (X_{k}^{t}(j)-X_{Male,i}^{t}(j))$$

where $X_{male,i}^{t+1}(j)$ represents the position of the i-th stag in the Male population at generation t + 1 on the j-th dimension; $X_{male,i}^{t}(j)$ represents the position of the i-th stag in the Male population at generation t on the j-th dimension; $X_{k}^{t}(j)$ represents the position on the j-th dimension of an individual randomly selected from the population; α rand is a random number in the interval [0, 1], used to control the proportion of inheritance from the randomly selected individual:

(3) $$X_{Female,n}^{t+1}(j)=X_{Female,n}^{t}(j)+\beta \times (X_{h}^{t}(j)-X_{Female,n}^{t}(j))+\gamma \times (X_{r}^{t}(j)-X_{Female,n}^{t}(j))$$

where $X_{Female,n}^{t+1}(j)$ denotes, on the j-th dimension, the location of the nth female individual within the female subpopulation at the (t + 1) to generation; $X_{Female,n}^{t}(j)$ represents the position of the n-th female in the Female population at generation t on the j-th dimension; $X_{h}^{t}(j)$ represents the position of the stag in the family to which the n-th female belongs at generation t; $X_{r}^{t}(j)$ represents the position of a stag randomly selected from other families at generation t; β and γ are random values in the interval [0, 2], controlling the genetic weights from the current family’s stag and an external stag, respectively.

## 3. Improved Elk Herd Optimiser

To further improve the performance of EHO, this section proposes an Improved Elk Herd Optimiser (IEHO). The corresponding pseudocode is outlined in the second algorithm (Algorithm 2).
**Algorithm 2: IEHO****Given**: number of individuals in the population: EHS, Problem dimensionality: D, Lower limit of the search range: lb, Upper limit of the search range: ub, Maximum number of iterations: Max_iter, Group size: G, Number of stags: B **Output**: Optimal solution and its fitness value1.  Variable initialization (EHS, D, ub, lb, Max_iter, G, B)2.  Randomly generate an initial population X containing N individuals within the domain3.  Obtain the fitness *f(Xi)* for Xi by evaluating the objective function4.  t = 15.  For t = 1 to Max_iter do6.          Rank the entire population according to fitness7.  //Rutting phase8.  Use Section 3.1 to divide the population into stags, females, and free individuals9.      //Calving phase10.      For each stag individual $x_{j}^{t}$ do11.                    $x_{j}^{t+1}$ ← Update position according to Section 3.2.112.    end For 13.        For each female individual $x_{j}^{t}$ do14.                    $x_{j}^{t+1}$ ← Update position according to Section 3.2.215.        end For16.        For each free individual $x_{j}^{t}$ do17.                       $x_{j}^{t+1}$ ← Update position according to Section 3.2.318.        end For19.      t = t + 120.end For21.Output the global optimal solution.

### 3.1. Improved Rutting Phase

#### 3.1.1. Analysis of the Rutting Phase of EHO Algorithm

**Figure 1:**
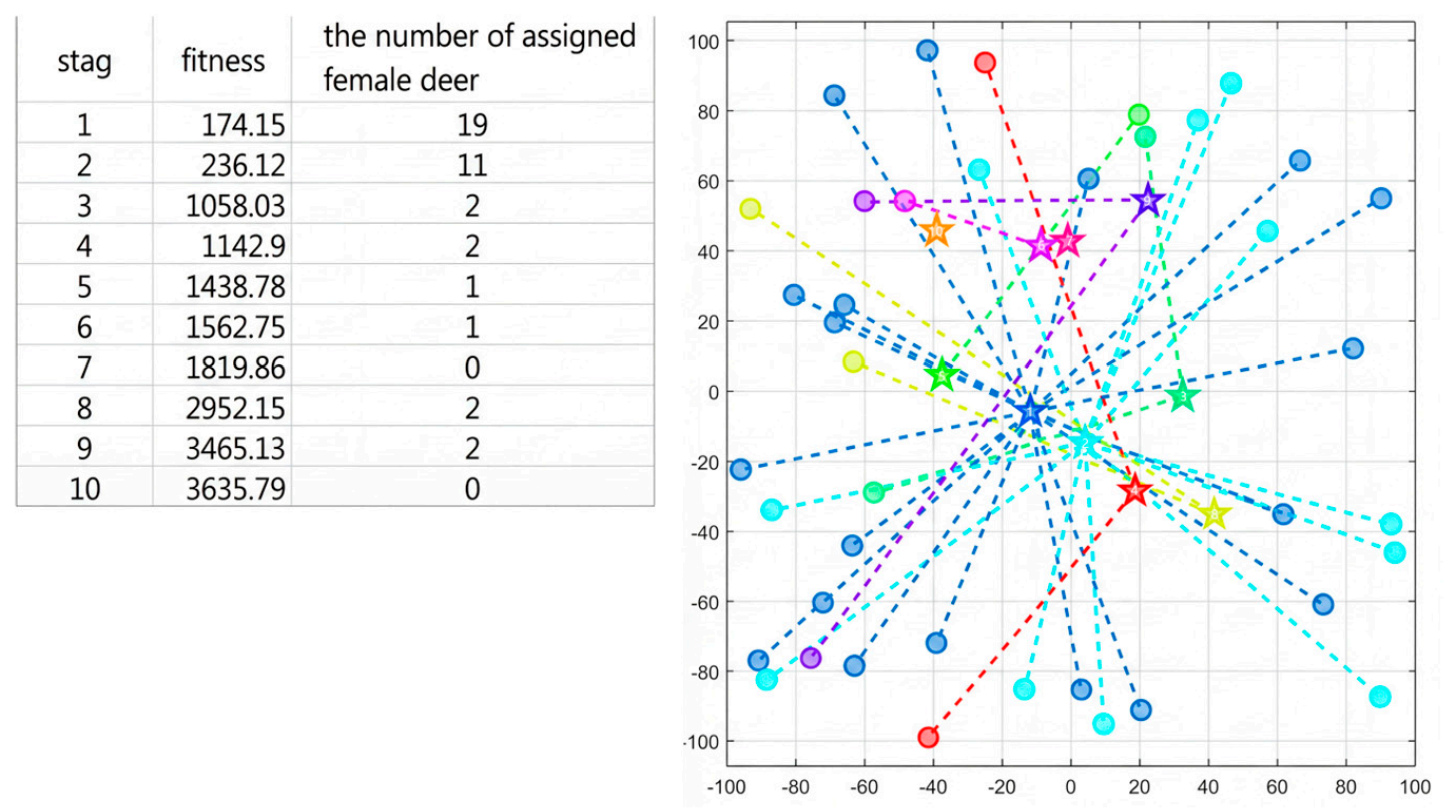
Early−stage family distribution.

**Figure 2:**
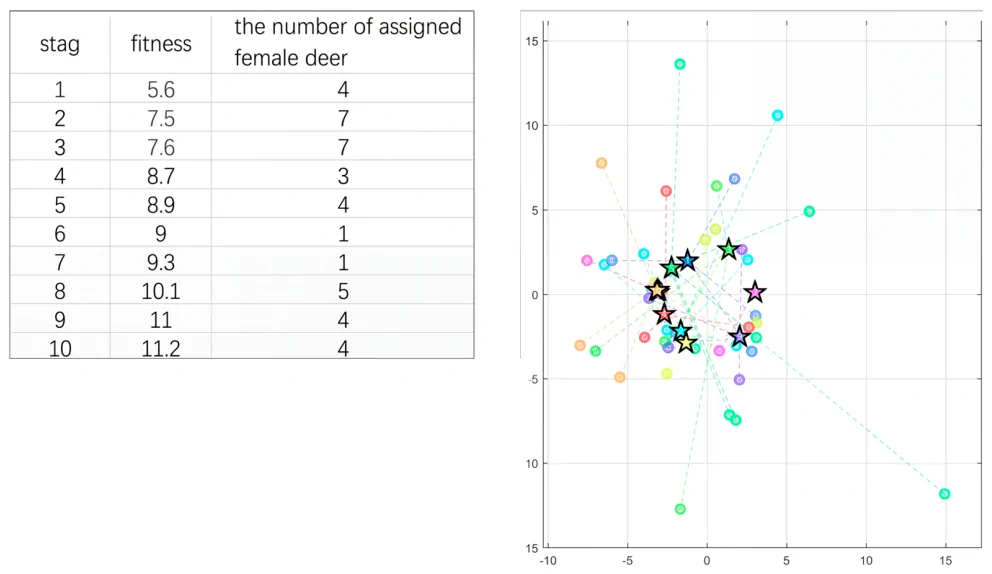
Late−stage family distribution.

As described in Section 2.1, the rutting phase of the EHO algorithm actually includes two main operations: First, determine the top |EHS × Br| individuals with better fitness as stags, and the rest as females; second, each female is assigned to the family of a corresponding stag using a roulette wheel selection mechanism. The subsequent calving phase performs individual updates on a family basis. The quality of the stag population and the rationality of family formation determined during the rutting phase directly affect the subsequent individual update results, thus influencing both the rate at which the algorithm converges and the precision of its final solution. During the initial phase of the search process, as illustrated in this family distribution diagram (Figure 1), for a population of 50 individuals, according to the rutting phase of the EHO algorithm, the top 10 individuals with better fitness are determined as stags, marked as “
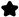
” in the Figure, and other “○” marks with the same colour as the “
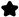
” represent individuals belonging to the same family. In the early stage, as shown in the family distribution (Figure 1), stags are relatively clustered, and the distribution of females assigned to each stag is very random, with large differences in family sizes. In the later stage, as shown in the family distribution (Figure 2), the fitness of stag M1 is better than that of stag M8, but stag M1 is assigned fewer females than stag M8, indicating that stags with better fitness are not necessarily assigned more females.

A thorough investigation identifies the root causes of the aforementioned deficiencies during EHO’s rutting stage. Firstly, in the initial search phase, EHO directly designates fitter individuals as stags, which improves the convergence speed to some extent. However, as shown in the left part of Figure 1, these stags with better fitness values often show high regional clustering. If these individuals are located at certain suboptimal peaks, using them as guides to direct the evolution of other individuals increases the risk of the search process being trapped in local optima.

Secondly, this roulette wheel selection mechanism (Formula (1)) is essentially a random probability drawing method based on fitness proportion. During the algorithm’s initial phase, the population members are relatively dispersed, and the disparities in fitness are substantial. When the fitness of an excellent stag is significantly higher than that of other individuals, this mechanism can assign more reproduction opportunities to it with a higher probability. However, in the later stage of the algorithm, individuals gradually converge, and fitness differences amongst individuals decrease. At this time, the inherent random fluctuations of the roulette wheel selection mechanism may cause stags with better fitness to obtain fewer reproduction opportunities owing to chance factors, whereas stags with lower fitness attract more females. This selection bias will prevent excellent stags from attracting enough females, thus limiting effective population updates and ultimately severely affecting the algorithm’s convergence rate and solution precision.

Thirdly, during EHO’s rutting phase, all female individuals are forcibly assigned to a family. However, some females are far from the stag of their own family or even from stags of other families. Forcing these individuals to rely on these stags for guidance makes all individuals in the population too similar to the relatively clustered stags, losing the diversity that these females originally provided to the population, greatly reducing the possibility of exploring other potentially advantageous regions and increasing the risk of the algorithm falling into local optima. In addition, each female can only be assigned to a single family, which means that during the entire iterative process, the female mainly follows the learning of the stag of her family, and her evolutionary direction is severely limited by the guidance of that stag. Even if better genetic information exists in other families, it cannot be utilised, exacerbating the loss of population diversity.

#### 3.1.2. IEHO Proposal of the Rutting Phase of IEHO Algorithm

In light of the preceding discussion, aiming to better reconcile how accurately and how quickly the algorithm converges, a novel rutting phase integrating fitness quality and spatial distribution information is designed, as detailed below.

Step 1: Determine the population’s fittest member as a stag and add it to the stag population, and form a candidate set from the other top 20% high-quality individuals based on fitness.

Step 2: Compute the Euclidean distance separating every candidate set member from all members of the stag herd. Select the minimum of the Euclidean distances from each candidate individual to the stag population as the distance from that individual to the stag population. Move the individual with the largest distance from the candidate set into the stag population.

Step 3: Repeat Step 2 until the number of individuals in the stag population reaches |0.5 × Br × EHS|.

Step 4: For each stag in the stag population, select the k individuals closest to it (generally, k is set to 7, which can also be adjusted according to the specific optimisation problem) to form a family together with it. Individuals not assigned to any family form a “free individual” group.

**Figure 3:**
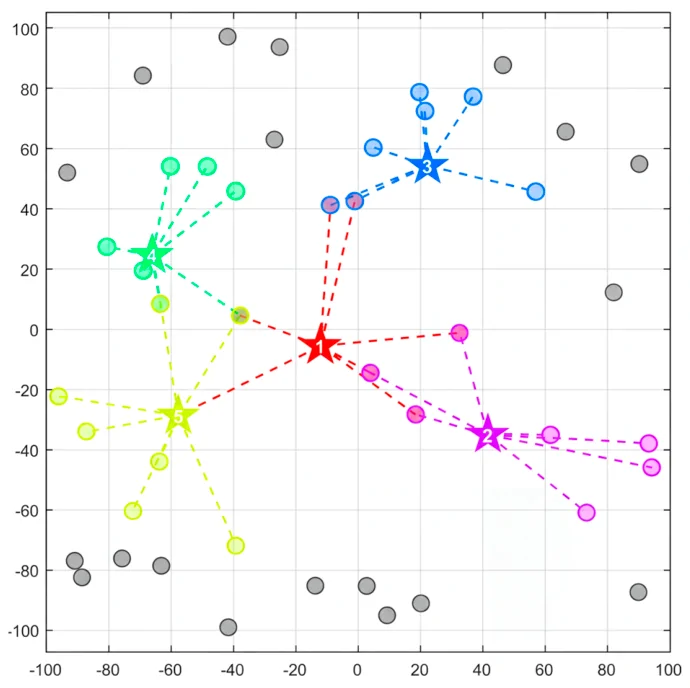
Early−stage improved family distribution.

**Figure 4:**
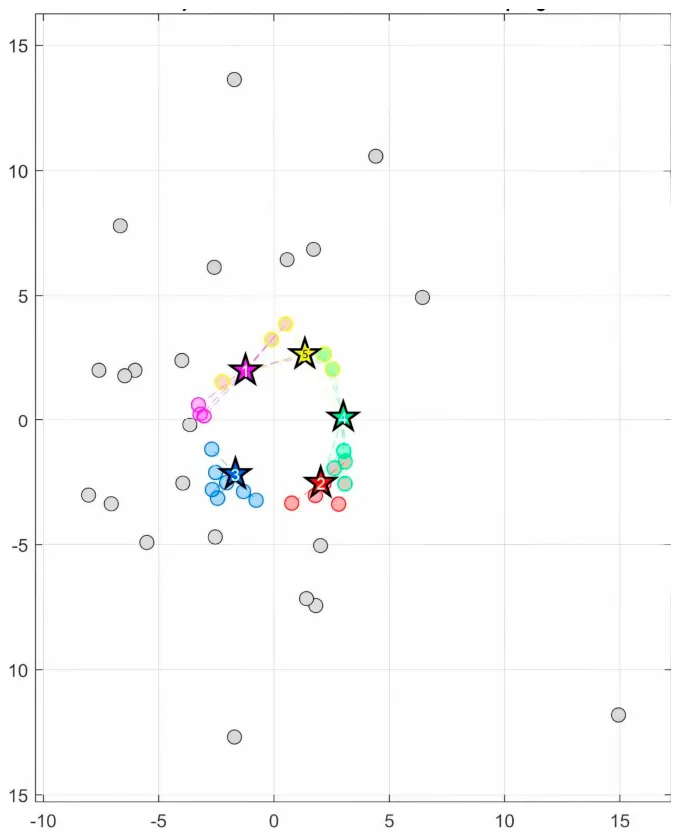
Late−stage improved family distribution.

According to the above rutting phase, the family assignments of individuals in the population are redistributed, with results shown in Figure 3 and Figure 4. In the early stage of the algorithm, stags are relatively scattered in the search space, all females are relatively close to stags, and some females belong to the families of two stags. In the later stage, stags can be assigned fixed females.

Compared with the rutting phase in Section 2.1, the improved rutting phase introduced herein offers the following merits: Firstly, except for that member with optimal fitness selected as a stag, the remaining stags are chosen from some individuals with relatively good fitness using the maximin Euclidean distance criterion. Unlike the original method of selecting stags on the basis solely of fitness values, although the fitness performance of these stags is slightly worse, the retention of the best fitness individual ensures the algorithm’s convergence direction. The maximin Euclidean distance considers the spatial distribution of individuals, making stags as dispersed as possible in different regions of the search space, evading the defect of falling into local optima caused by excessive clustering in the original method. At the same time, the number of stags is adjusted to |0.5 × Br × EHS|, retaining only representative stags in the region and avoiding the clustering between families caused by too many stags in the original algorithm. Families become more dispersed, each searching within its own space, increasing the possibility of finding more and better solutions. Secondly, during family assignment, the roulette wheel selection mechanism is abandoned in favour of a distance-based grouping mechanism, in which stags directly select several individuals that are spatially close to them to form families. Compared with the roulette wheel selection mechanism, this strategic grouping method, based on distance to select individuals to form families, avoids the problem of insufficient assignment to individuals with better fitness due to random fluctuations of roulette wheel selection in the later stage, when differences between individuals are small. The family size is uniformly set to a fixed value, preventing uneven allocation of search resources due to there being too many or too few members in a particular family. Thirdly, in contrast with the original rutting phase in which all females must be forcibly assigned to a family, this section does not assign any family to those individuals that are geographically far from stags, suppressing the impact of their overly scattered spatial distribution on the search performance of other family members when forced to join a family. New individual update mechanisms are designed for these individuals in the subsequent calving phase, enhancing the algorithm’s universality in solving different optimisation problems.

### 3.2. Improved Calving Phase

The EHO algorithm relies on the calving phase for individual updates. To better balance how rapidly and how precisely the algorithm converges, the present section comprehensively improves the original paternal inheritance pattern for stags and the maternal reproduction pattern for females in the EHO algorithm’s calving phase, and it designs new individual update mechanisms for free individuals, as detailed below. It is important to emphasise here that unlike the selection method of the original EHO algorithm, which merges the newly generated population with the original population and takes the better half as the iterative population, this section uses a one-to-one elite retention method between new individuals and original individuals to determine the iterative individuals and avoid excessively fast convergence.

#### 3.2.1. Improved Paternal Inheritance Pattern for Stags

In the calving phase, the stag of each family updates its position by randomly selecting an individual from the population according to Formula (2). This approach has a certain degree of randomness. Although it maintains population diversity to some extent, it reduces the guiding role of dominant individuals and greatly slows down the algorithm’s convergence rate. Moreover, this step factor α is a random number between [0,1]. In the early iteration, the population is relatively scattered, and a small step size prevents individuals from converging quickly. In the later stage of evolution, when the population is already close to the optimal region, this random step size prevents fine-grained search. That is, this random step factor cannot meet the requirements of different evolutionary stages for step size.

Given this, to better highlight the directional guidance of stags and effectively balance the convergence speed and population diversity of the algorithm, the paternal inheritance pattern for stags in each family is improved, as shown in Formula (4):

(4) $$X_{Male,i}^{t+1}=\left\{\begin{array}{l} -X_{Male,i}^{t+1},\;if\;rand<Pob1^{t}\;\&\;\&\;f(-X_{Male,i}^{t+1})<f(X_{Male,i}^{t+1})\\ X_{Male,i}^{t+1},\;else \end{array}\right.\;$$

where $Pob1^{t}$ is the probability of opposition-based learning at generation t, as specifically shown in Formula (5); $X_{Male,i}^{t+1}$ and $-X_{Male,i}^{t+1}$ represent the new stag generated according to Formula (6) and its opposite individual, respectively:

(5) $$Pob1(t)=p_{\min}+(p_{\max}-p_{\min})\times \frac{t}{T}$$

where $p_{\min}$ and $p_{\max}$ are the lower and upper bounds of the opposition-based learning probability (generally set to 0.1 and 0.5 for good results, also adjustable according to the specific optimisation problem), and t and T represent the current iteration number and the maximum iteration number, respectively:

(6) $$X_{Male,i}^{t+1}(j)=X_{Male,i}^{t}(j)+g\times (X_{Male,i}^{t}(j)-X_{b}^{t}(j))+h\times (X_{r}^{t}(j)-X_{Male,i}^{t}(j))$$

where $X_{Male,i}^{t+1}(j)$ represents the position of the new calf produced by the i-th stag in the Male population at generation t + 1 on the j-th dimension; $X_{b}^{t}(j)$ represents the position of a randomly selected female from the family of the i-th stag at generation t; $X_{r}^{t}$ represents a stag randomly selected from other families; The dynamic learning parameters h and g are calculated as shown in Formulas (7) and (8):

(7) $$h=(0.5-sigmoid\times 0.2)\times r_{g}$$

(8) $$g=(0.5+sigmoid\times 0.3)\times r_{g}$$

where the ranges of sigmoid and $r_{g}$ are as shown in Formulas (9) and (10):

(9) $$sigmoid=\frac{1}{1+e^{-10\times (t/T-0.5)}}$$

(10) $$r_{g}=0.9+0.2\times rand$$

In contrast with the conventional approach of randomly selecting an individual from the population for exchange learning in Formula (2), the stag in the proposed Formula (Formula (6)) learns jointly from stags of other families and a female within its own family. Learning from other stags breaks the information barrier between families in the original algorithm, allowing high-quality genes to spread across the entire population and keeping the population diverse whilst preserving the evolutionary direction of the search. Introducing a female into its own family allows the stag to conduct fine-grained local exploration within that family’s local region, ensuring convergence direction. That is, the learning pattern of Formula (6) enables the stag to absorb local information within the family for refined exploitation whilst sensing the search progress of other elite stags for collaborative evolution, solving the problems of a single information source and blind search direction in the original algorithm.

As shown in Formulas (7)–(10), $r_{g}$ ranges between [0.9, 1.1], and sigmoid is within [0,1]. The dynamic learning factor h controls the degree of learning from other stags, thereby regulating the extent of global exploration. It dynamically decreases from 0.5 to 0.3 as the iteration proceeds. Meanwhile, the dynamic learning factor g controls the degree of communication with the female in the family, thus managing the level of local exploitation. It dynamically increases from 0.5 to 0.8. In the early stage of evolution, h and g are roughly equal, balancing the algorithm’s global exploration and local exploitation capabilities. As the iteration proceeds, population individuals become closer to the theoretical optimum, h decreases, and g increases, making stags focus more on local exploitation to avoid missing the theoretical optimum. The smooth and gradual transition of h and g meets the requirements of the algorithm for the evolution of dominant stags at different stages, achieving adaptive balance between exploration and exploitation.

After the stag is updated in accordance with Formula (6), opposition-based learning is performed with the probability shown in Formula (5) to generate a new stag. Opposition-based learning can provide new exploration positions for the population, supplement population diversity and, to some extent, enhance the algorithm’s ability to escape local optima. As presented in Formula (5), this probability gradually increases from 0.1 to 0.5 as the iteration proceeds. During the initial phase of the evolutionary process, the population exhibits a relatively high level of diversity; a smaller probability allows a small number of individuals to perform opposition-based learning, exploring new positions without destroying the original evolutionary direction. In the later stage of evolution, population diversity may be insufficient; the probability increases but not excessively, providing more diversity to the population and helping escape local optima whilst avoiding excessive introduction of opposition-based learning that could cause severe oscillations in the solution space or even degenerate into pure random search.

#### 3.2.2. Improved Maternal Reproduction Pattern for Females

In natural ecosystems, female deer individuals typically mate not only with males from their own familial group but also engage in gene exchange with males from other groups to optimise offspring quality. Equation (3) in the original EHO algorithm was devised to simulate this biological phenomenon; however, it harbours two critical deficiencies. First, the learning objects of females are confined exclusively to male deer, despite the fact that the female subpopulation substantially outnumbers its male counterpart. This asymmetric learning mechanism leads to an overly narrow spectrum of search directions and impoverishes information sources, thereby accelerating the premature erosion of population diversity. Second, the learning step size is governed entirely by independent stochastic parameters β and γ with values restricted to the interval [0, 2], which introduces considerable uncertainty into the evolutionary direction and impedes the formation of an effective collective search force. This stochasticity becomes particularly pronounced during the later stages of optimisation, where severe random-walk behaviour emerges and substantially compromises convergence performance.

To address the above issues, this section designs a novel individual update mechanism for female deer individuals that integrates multiple strategies in a synergistic manner, as formally presented in Formula (11). This mechanism introduces systematic improvements from four aspects: mate selection, mutation strategy, adaptive step size, and dimensional crossover:

(11) $$X_{Female,n}^{t+1}(j)=\left\{\begin{array}{l} A^{t+1}(j),\;if\;rand<CR||j=j_{rand}\\ X_{deer}^{t}(j),\;else \end{array}\right.\;$$

where $X_{Female,n}^{t+1}(j)$ represents the position of the n-th female individual at generation t + 1 on the j-th dimension; rand is a random number drawn from a uniform distribution over the interval [0, 1], independently generated for each dimension; $j=j_{rand}$ is a randomly selected integer from the set {1,2…,D}, where D is the problem’s dimensionality. This ensures that at least one dimension undergoes crossover, preventing the generation of an individual that is completely identical to the stag; CR is the crossover probability, which controls the probability of inheriting the gene from the intermediate offspring on each dimension. Generally, setting CR to 0.8 yields satisfactory results, though it can also be adjusted according to the specific problem under consideration; $X_{deer}^{t}(j)$ represents the value on the j-th dimension of the mating partner selected by the female according to Formula (12) at generation t. $A^{t+1}(j)$ represents the value of the intermediate offspring generated by Formula (13) on the j-th dimension at generation t. This variable serves as the primary carrier of new genetic information.

The improved mate selection strategy is as follows:

(12) $$X_{deer}^{t}(j)=\left\{\begin{array}{l} X_{nowdeer}^{t}(j),\;if\;rand<0.5\\ X_{otherdeer}^{t}(j),\;else \end{array}\right.\;$$

$X_{nowdeer}^{t}(j)$ denotes the position on the j-th dimension of the stag belonging to the same family as the n-th female at generation t. $X_{otherdeer}^{t}(j)$ denotes the position on the j-th dimension of a stag randomly selected from other families at generation t. As shown in Formula (12), when the random number rand < 0.5, a female individual selects the male deer $X_{nowdeer}^{t}(j)$ from her own familial group as the mating partner. Owing to the relatively close spatial proximity among family members during the improved rutting phase, this selection strategy guides female individuals toward local exploitation within the current favourable regions, thereby accelerating population convergence. Conversely, when rand ≥ 0.5, the female selects a male $X_{otherdeer}^{t}(j)$ from a different familial group to facilitate cross-family gene exchange. This cross-family mating mechanism effectively breaks down the information barriers among distinct families in the original algorithm and promotes the dissemination of superior genetic information among female individuals.

A differential intermediate offspring generation strategy:

To enhance the exploration capability of the population and augment the diversity of candidate solutions, we design Formula (13) to generate the intermediate offspring $A^{t+1}(j)$. This strategy constitutes a substantive improvement over the original formulation of Formula (3):

(13) $$A^{t+1}(j)=phi1\times X_{Female,n}^{t}(j)+(1-phi1)\times X_{deer}^{t}(j)+phi2\times (X_{r1}^{t}(j)-X_{r2}^{t}(j))$$

where $X_{Female,n}^{t}(j)$ represents the position of the n-th female individual at generation t on the j-th dimension; $X_{deer}^{t}(j)$ is the stag individual selected by Formula (12); $X_{r1}^{t}(j)$, $X_{r2}^{t}(j)$ represent two distinct individuals randomly selected from the population at generation t, with indices r1 and r2 satisfying r1 ≠ r2 ≠ n; parameters phi1 and phi2 are adaptive step-size factors, which are dynamically adjusted as shown in Formulas (14) and (15), respectively.

Adaptive step-size control based on truncated Cauchy distribution:

Given that the step-size factors phi1 and phi2 are pivotal to balancing exploration and exploitation, we propose a dynamic adjustment mechanism based on population diversity feedback:

(14) phi1=Cauchy(μ=1,γ=0.2)

(15) $$phi2=Cauchy(\mu =Phi_{bar},\gamma =0.3)$$

In this context, Cauchy denotes a random number sampled from the truncated Cauchy distribution. The Cauchy distribution is chosen because its heavy-tailed property allows for occasional large jumps, which is beneficial for escaping local optima. The distribution is truncated to constrain the generated values within a reasonable range, ensuring algorithmic stability. μ and $Phi_{bar}$ are the location parameters of the Cauchy distribution, which determine the central tendency of the generated step-size values. γ denotes the scale parameter, governing the degree of dispersion of the step-size factors about their location parameters. The location parameter $Phi_{bar}$ is not fixed but is adaptively adjusted according to the current population diversity, and it is updated according to Formula (16):

(16) $$Phi_{bar}=0.5+0.5\times (1-\eta )$$

where η is the current population diversity based on median distance, as specifically shown in Formula (17):

(17) $$\eta =min\left(\frac{current\_diversity}{DIV_{0}},1\right)$$

(18) $$current\_diversity=\frac{1}{N}\sum_{i=1}^{N}(\frac{1}{D}\sum_{j=1}^{D}|X_{i,j}-median(X_{.,j})|)$$

Here, current_diversity denotes the current population diversity measured based on median distance, as formally defined in Formula (18), where N is the population size and D is the dimensionality of the optimisation problem; $X_{i,j}$ denotes the j-th dimension of the i-th individual; $median(X_{.,j})$ represents the median value of all individuals on the j-th dimension; $DIV_{0}$ is the initial population diversity based on the median distance of the population. The underlying principle of this adaptive mechanism is described as follows: When the population diversity is high (current_diversity is large, which typically occurs in the early exploration stage), η becomes larger while the location parameter $Phi_{bar}$ becomes smaller, resulting in smaller generated values. This facilitates local exploitation around the female individuals in the early stage of the algorithm, thereby enhancing the population’s convergence speed. Conversely, when the population diversity decreases, the location parameter $Phi_{bar}$ increases, leading to larger generated values of phi2. This is beneficial in the later stage of the algorithm, when population diversity declines, as it helps individuals escape from local optima.

Dimensional crossover and elite retention:

Finally, the newly generated intermediate offspring $A^{t+1}(j)$ undergoes a dimensional crossover operation with its selected mate $X_{deer}^{t}(j)$ according to Formula (11). Specifically, for each dimension, the offspring inherits the dimensional value from the intermediate offspring $A^{t+1}(j)$ with probability CR and inherits that from the selected mate $X_{deer}^{t}(j)$ with probability 1−CR. This mechanism ensures that the algorithm robustly preserves and integrates superior genetic information derived from different strategies in each generation, thereby effectively avoiding the bias that may arise from relying on any single strategy.

Unlike the maternal inheritance method within the EHO algorithm, the enhanced maternal inheritance pattern described in this section possesses the following strengths.

(1) Females learn from the stag of their own family with probability 0.5 and from a stag of another family with probability $0.5\times (\frac{1}{\left|0.5\times Br\times EHS\right|-1})$. The probability of learning from the stag from their own family is significantly higher than that from stags from other families. According to the mechanism in Section 3.1, females and stags within a family are relatively close geographically; hence, this learning mechanism ensures local exploitation of individuals to a greater extent. Each family maintains local exploitation well in its own space, thereby ensuring global exploration of the population. Learning from stags of other families also increases communication between families to some extent, facilitating the exploration of new search regions.

(2) As shown in Formula (13), the female not only continues to learn from the stag of its own or other families but also incorporates pairwise learning between two randomly selected agents from the swarm, further increasing communication between individuals, enhancing exploration of new regions and improving population diversity.

(3) Using $phi1\times X_{Female,n}^{t}(j)+(1-phi1)\times X_{deer}^{t}(j)$ as a base vector and the truncated Cauchy distribution parameter phi1, the generated intermediate calf retains the genes of the female individual $X_{Female,n}^{t}(j)$ whilst introducing the genes of the stag $X_{deer}^{t}(j)$, accelerating the convergence speed of the female towards the stag. During population evolution, as differences between population individuals decrease and population diversity declines, parameter phi2 is set to increase gradually with $Phi_{bar}$, where b increases from 0.5 to 1 as the diversity of the population decreases. Through $phi2\times (X_{r1}^{t}(j)-X_{r2}^{t}(j)$ increasing with iteration, the communication between the base vector and random individuals $X_{r1}^{t}(j)$, $X_{r2}^{t}(j)$ is enhanced, expanding the exploration of new regions for individuals, preventing convergence homogeneity due to rapid convergence, increasing population diversity, and reducing the possibility of falling into local optima.

(4) From Formula (11), by crossing the intermediate calf $A^{t+1}(j)$ with the stag, stag genes are further introduced into the intermediate calf, increasing the speed of convergence towards stags whilst retaining female evolution and raising the possibility of generating better individuals.

#### 3.2.3. Update Method for Free Individuals

In summary, stags are a number of excellent individuals integrating fitness and distance, and females are individuals relatively close to each stag. According to the evolution methods in Section 3.2.1 and Section 3.2.2, both the stag population and female population focus on exploration near the relatively better stag regions, improving the overall convergence speed of the algorithm while taking into account global search to some extent. For free individuals far from stags, they are not all poor in fitness; there are still some high-quality individuals with good fitness performance. To fully balance the global exploration and local exploitation capabilities of the population, different evolution strategies are designed for individuals with different fitness performances. Specifically, free individuals ranked in the top M of the overall population fitness (M is set to 30% in the research), called high-quality free individuals, are utilised for local exploitation near high-fitness individuals, facilitating expedited overall algorithmic convergence. Other free individuals with poorer fitness performance, called low-quality free individuals, are used for global exploration to provide more population diversity. The specific evolution strategies are as follows.

(1) Evolution strategy for high-quality free individuals

This section designs an evolution strategy for high-quality free individuals as shown in Formula (19):

(19) $$X_{Fb,m}^{t+1}(j)=X_{Fb,m}^{t}(j)+g\times (X_{Fb,m}^{t}(j)-X_{near}^{t}(j))+h\times (X_{Male,r}^{t}(j)-X_{Fb,m}^{t}(j))$$

where $X_{Fb,m}^{t+1}(j)$ represents the position of the m-th high-quality free individual at generation t + 1 on the j-th dimension; $X_{Fb,m}^{t}(j)$ represents the position of the m-th high-quality free individual at generation t on the j-th dimension; $X_{near}^{t}(j)$ represents the position on the j-th dimension of the individual closest to the m-th high-quality free individual at generation t; $X_{Male,r}^{t}(j)$ represents a randomly selected stag from the stag population on the j-th dimension.

(2) Evolution strategy for low-quality free individuals

Inspired by the mutation strategy in differential evolution, this paper designs the individual evolution mechanism for inferior free individuals, as shown in Equation (20), with the detailed formulation given in Equation (21). This strategy shares a similar evolutionary philosophy with the elitism-guided multi-population collaborative mechanism in the DEEM algorithm proposed by Machaček et al. [25]. However, we further introduce a truncated Cauchy distribution-based dynamic step-size control to accommodate the specific role of free individuals as follows:

(20) $$x_{Fd,o}^{t+1}(j)=\left\{\begin{array}{l} A^{t+1}(j),\;if\;rand<CR_{2}||j=j_{rand}\\ X_{c}^{t}(j),\;else \end{array}\right.\;$$

where $x_{Fd,o}^{t+1}(j)$ denotes the coordinate of the m-th high-quality free individual at generation t + 1 along dimension j; $X_{c}^{t}(j)$ denotes an individual randomly chosen from non-free individuals in generation t along dimension j; $CR_{2}$ is the crossover probability, generally set to 0.9 for good results, also adjustable according to the optimisation problem; $A^{t+1}(j)$ represents the result of learning from $X_{c}$ according to Formula (21):

(21) $$A^{t+1}(j)=\phi_{1}\times X_{Fd,o}^{t}(j)+(1-\phi_{1})\times X_{c}^{t}(j)+\phi_{2}\times (X_{r1}^{t}(j)-X_{r2}^{t}(j))$$

where $X_{r1}^{t}(j)$ and $X_{r2}^{t}(j)$ represent a randomly selected individual from the grouped individuals at generation t; $\phi_{1}=Cauchy(0.6,0.3)$ and $\phi_{2}=Cauchy(Phi_{bar},0.3)$ are Cauchy random numbers.

Compared with the EHO algorithm, the increased evolution strategy for free individuals in this section has the following advantages.

(1) Free individuals are divided into high- and low-quality free individuals on the basis of their fitness. High-quality free individuals evolve in accordance with Formula (19). Such individuals, based on themselves, learn jointly from the individual closest to them and other stags. Learning from the closest individual allows for more detailed exploitation around themselves to find better solutions, whilst learning from other stags spreads the excellent genes of stags in the population, improving population convergence speed whilst maintaining diversity. Low-quality free individuals evolve in accordance with Formula (21). This formula, based on themselves, takes a randomly selected individual from the already grouped individuals as guidance whilst communicating with two random individuals in the population. This evolution method allows low-quality free individuals to move over a wide range in the population to explore new regions, increasing the possibility of generating better solutions. These two evolution methods assign different tasks to two different types of individuals: high-quality free individuals intensify local exploitation to enhance algorithm convergence, while low-quality free individuals perform wide-range exploration in the region to maintain population diversity. The combination of the two tasks helps the algorithm achieve a dynamic balance between exploitation and exploration during the search process. At the same time, this distinction between family individuals and free individuals allows family individuals to perform more refined searches within their family regions, whilst free individuals absorb high-quality genes by learning from family individuals and explore and exploit on the basis of themselves to find better individuals. The cooperation between the two types of individuals enables the algorithm to achieve a better balance between convergence speed and population diversity.

(2) When evolving high-quality free individuals, to utilise their good genes, exploitation around their surroundings is needed to increase the possibility of finding better solutions. Therefore, this study uses the individual update formula shown in Formula (19). This formula can be summarised as ‘base vector + step1 × difference vector1 + step2 × difference vector2’. Difference vector1, the mutual learning between the current high-quality free individual and its nearest individual $X_{near}^{t}(j)$, is beneficial for exploration near the current high-quality free individual. Step1 increases from 0.5 to 0.8 as the number of iterations increases. In the later stage of evolution, when individuals are relatively clustered, it further increases the range of position change in the current high-quality free individual, expanding population diversity. Difference vector2, the mutual learning between the current high-quality free individual and a randomly selected stag, is advantageous for the current high-quality free individual to learn from stags in different directions, allowing it to accelerate convergence speed whilst hardly falling into local optima. Step2 decreases from 0.5 to 0.2, so that in the early stage when individuals are relatively scattered, a larger step size moves towards dominant individuals, thereby accelerating convergence speed; in the later stage, when individuals are relatively clustered, a smaller step size allows fine-grained local exploitation.

(3) When evolving low-quality free individuals, because of their poor fitness, they need to perform wide-range exploration within the population to find better solutions. Accordingly, this study uses the individual update formula shown in Formula (21). In this formula, the low-quality free individual uses $\phi_{1}\times X_{Fd,o}^{t}(j)+(1-\phi_{1})\times X_{c}^{t}(j)$ as the base vector and takes a randomly selected individual from the already grouped individuals $X_{c}^{t}(j)$ as guidance. Given that the grouped individuals are numerous and widely distributed, excessive concentration in a single direction is avoided, allowing the current low-quality free individual to explore in more directions to find better solutions. During population evolution, individuals gradually converge, making differences between individuals smaller, $\phi_{2}\times (X_{r1}^{t}(j)-X_{r2}^{t}(j)$. Based on the Cauchy distribution principle, as $\phi_{2}$ gradually increases with $Phi_{bar}$ (which increases from 0.5 to 1), the possibility of finding more and better solutions near the basis vector $\phi_{1}\times X_{Fb,m}^{t}(j)+(1-\phi_{1})X_{c}^{t}(j)$ is enhanced. Moreover, exchanging the basis vector with random individuals $X_{r1}^{t}(j)$ and $X_{r2}^{t}(j)$ in the population increases information sharing between the disadvantaged free individual and other individuals. This approach not only improves population diversity but also broadens the search directions of the disadvantaged free individual, thus increasing the likelihood that the disadvantaged free individual finds a better solution.

(4) To meet the specific role of low-quality free individuals in population evolution and maintain population diversity, this study performs dimensional crossover in Formula (20) and increases the crossover probability CR to 0.9. This method retains the genes of the intermediate calf to a great extent, allowing low-quality free individuals to explore freely in the population with a high probability of being unaffected by the genes of nongrouped individuals, increasing the possibility of individuals finding better solutions and maintaining the heterogeneity of the population.

## 4. Experimental Outcomes and Interpretation

To comprehensively evaluate the performance of the proposed IEHO algorithm, three sets of experiments are conducted in this section: (1) parameter sensitivity analysis; (2) validation of the effectiveness of each improvement strategy; and (3) performance comparison with state-of-the-art intelligent optimisation algorithms.

All experiments are carried out on the CEC2017 test suite, which consists of 29 benchmark functions, including unimodal functions, multimodal functions, and composite functions. Specifically, F1–F3 are unimodal functions with a single global optimum, which are used to evaluate the convergence capability of the algorithms. F4–F10 are multimodal functions with multiple local optima, which serve to examine the algorithms’ ability to escape from local optima. F11–F30 are hybrid and composition functions, which are employed to assess the overall comprehensive performance of the algorithms. All algorithms are implemented in MATLAB R2021a and run on a computer equipped with the Windows 10 operating system and an i5-7200U CPU.

### 4.1. Parameter Sensitivity Analysis

The proposed IEHO algorithm adds or modifies the following parameters based on the original EHO algorithm: Br, k, Pmin, Pmax, g, and h. When investigating the influence of a specific parameter on the performance of IEHO, that parameter is set to five different values while keeping the other parameters unchanged. In all experiments, the population size is set to N = 50, the problem dimension is D = 30, and the maximum number of function evaluations is max_FE = 100,000. The other parameter settings are as follows:

(1) When examining the effect of Br on the performance of IEHO, Br is set to 0.04, 0.1, 0.2, 0.3, and 0.4, respectively, while the other parameters are fixed as k = 7, Pmin = 0.1, and Pmax = 0.5, g = [0.5–0.8], h = [0.5–0.3].

(2) When examining the effect of k on the performance of IEHO, k is set to 5, 6, 7, 8, and 9, respectively, while the other parameters are fixed as Br = 0.1, Pmin = 0.1, and Pmax = 0.5, g = [0.5–0.8], h = [0.5–0.3].

(3) When examining the effect of Pmin and Pmax on the performance of IEHO, these two parameters are set to the following five groups of values: 0 and 0.4, 0.1 and 0.5, 0.2 and 0.6, 0.3 and 0.7, and 0.4 and 0.8, while the other parameters are fixed as Br = 0.1 and k = 7, g = [0.5–0.8], h = [0.5–0.3].

(4) To examine the influence of the range of parameter g on the performance of the IEHO algorithm, the value range of g was set to the following five groups: [0.3–0.6], [0.4–0.7], [0.5–0.8], [0.6–0.9], and [0.7–1]. The other parameters were fixed as follows: Br = 0.1, k = 7, Pmin = 0.1, Pmax = 0.5 and h = [0.5–0.3].

(5) To examine the influence of the range of parameter h on the performance of the IEHO algorithm, the value range of h was set to the following five groups: [0.3–0.1], [0.4–0.2], [0.5–0.3], [0.6–0.4], and [0.7–0.5]. The other parameters were fixed as follows: Br = 0.1, k = 7, Pmin = 0.1, Pmax = 0.5 and g = [0.5–0.8].

All experiments are independently run 30 times on the CEC2017 test suite. For each run, the average of the optimal values obtained upon reaching the same maximum number of function evaluations is recorded. The detailed results are presented in Table 1, Table 2, Table 3, Table 4 and Table 5, where the best values are highlighted in bold. To compare the performance of the algorithm under different parameter settings, this study conducts the Friedman rank-sum test. The results are shown in the last two rows of Table 1, Table 2, Table 3, Table 4 and Table 5. A smaller average rank indicates better overall performance of the algorithm under that parameter setting. From Table 1, in terms of the number of test functions on which the optimal solution is achieved, when Br = 0.1, IEHO achieves the best performance on 16 out of 29 test functions. When Br is set to 0.04, 0.2, 0.3, and 0.4, the number of functions on which the optimum is achieved are 2, 0, 4 and 6, respectively. On function F22, the performance differences amongst all parameter settings are relatively small. Overall, based on the number of functions on which the optimum is achieved, IEHO performs best when Br = 0.1, whilst the performance at Br = 0.3, 0.4 is comparable. In the same analysis for Table 2, when k = 7, IEHO achieves the best performance on 14 out of 29 test functions. When k is set to 5, 6, 8 and 9, the number of functions on which the optimum is achieved are 3, 1, 6 and 6, respectively. In the performance at k = 8, 9 is similar, whereas the performance at k = 6 is relatively unsatisfactory. From Table 3, when the interval of Pmin and Pmax is set to 0.1–0.5, IEHO reaches the best performance on 16 out of 29 test functions. When the intervals are set to 0–0.4, 0.2–0.6, 0.3–0.7 and 0.4–0.8, the numbers of functions on which the optimum is achieved are 5, 2, 2 and 0, respectively. On functions F3, F22, and F27, the performance differences amongst the various interval settings are negligible. Overall, the algorithm performs best when the interval of Pmin and Pmax is set to 0.1–0.5. From Table 4, it can be seen that when the value interval of parameter g is [0.5–0.8], IEHO achieves the optimal performance on 14 test functions, while the intervals [0.3–0.6], [0.4–0.7], [0.6–0.9], and [0.7–1] correspond to 1, 1, 3, and 8 optimal functions, respectively. For functions F22 and F25, the performance differences across all intervals are relatively small. Using the number of optimal solutions as the evaluation criterion, the interval [0.5–0.8] is the optimal choice. Finally, as shown in Table 5, when the interval of parameter h is set to [0.5–0.3], IEHO achieves the optimal performance on 17 test functions, outperforming the intervals [0.3–0.1], [0.4–0.2], [0.6–0.4], and [0.7–0.5], which yield 4, 1, 3, and 3 optimal functions, respectively. For function F22, the performance differences across all intervals are negligible. Overall, the algorithm achieves its best performance when h is taken within the interval [0.5–0.3].

In summary, the IEHO algorithm is relatively sensitive to parameters Br, Pmin, Pmax, and h, while its sensitivity to h and k decreases slightly. Finally, according to the Friedman rank-sum test results presented in the last two rows of Table 1, Table 2, Table 3, Table 4 and Table 5, it can be observed that when Br = 0.1, k = 7, Pmin = 0.1, Pmax = 0.5, g = [0.5–0.8], and h = [0.5–0.3], the IEHO algorithm achieves the smallest average rank, indicating that the algorithm exhibits the optimal optimisation performance under these parameter settings. If better performance is desired for a specific optimisation problem, further fine-tuning of the above parameters through multiple trials can also be conducted.

### 4.2. Experimental Validation of Each Improvement Strategy

The IEHO algorithm introduces three major improvements over the original EHO framework. To thoroughly investigate the effectiveness of each individual improvement strategy, this study systematically constructs three IEHO variants by selectively removing one corresponding improvement at a time. These variants are designated as follows: IEHO1, which is obtained by removing the improved estrus phase described in Section 3.1.2 from IEHO; IEHO2, which excludes the improved father–son inheritance phase detailed in Section 3.2.1; and IEHO3, which eliminates the improved mother–son breeding phase presented in Section 3.2.2. The performance of these three variants, along with the complete IEHO algorithm, is then comparatively evaluated using the CEC2017 benchmark.

To ensure fairness in comparison, the population size of all algorithms is set to N = 50, the dimensionality of the test functions is D = 30, and the maximum number of function evaluations is max_FE = 100,000. The other parameter settings of each algorithm are presented in Table 6. To avoid the contingency caused by a single run, each algorithm is independently executed 30 times on each test function. Table 7 reports the experimental results of all algorithms on 29 test functions in 30 dimensions, where the values before the “±” sign represent the mean, and those after represent the variance. Moreover, for the same test function, the results of the improved algorithms that remove one enhancement strategy and are inferior to IEHO are highlighted in bold. To further examine the differences between IEHO and the other improved algorithms, the Wilcoxon rank-sum test with a significance level of 5% and the Friedman test are adopted between IEHO and each comparison algorithm, and the detailed results are shown in Table 8 and Table 9, respectively. In these Tables, the symbols “+”, ”−” and “=” denote the outcomes of the Wilcoxon rank-sum test.

The underlying principles of these two statistical tests are as follows. The Wilcoxon rank-sum test first pools the samples from two algorithms and sorts them in ascending order, then converts the sample values into their corresponding ranks. By comparing the ranks between the two samples, if the larger ranks are predominantly concentrated in one sample, it indicates a notable difference between the two algorithms. Specifically, when the resulting *p*-value is less than 0.05 and the mean value is superior to that of IEHO, the comparison algorithm is considered to outperform IEHO, denoted by “+”; otherwise, the opposite is indicated by “−”. When the *p*-value exceeds 0.05, the difference between the variant and IEHO is deemed statistically insignificant, denoted by “=”. As for the Friedman test, it computes the average rank of the samples for each algorithm; a smaller mean rank implies better overall performance across all test functions.

From Table 7, compared with the results of the IEHO algorithm, the numbers of functions on which IEHO1, IEHO2 and IEHO3 perform worse are 25, 19 and 28, respectively. Table 8 indicates performance differences between each improved algorithm and the IEHO algorithm. IEHO1 outperforms IEHO on three functions but underperforms on 18, with comparable results on the remaining eight; IEHO2 is superior on only one function, is inferior on seven, and shows no significant difference on 21; IEHO3 exhibits the clearest disadvantage, underperforming on 27 functions and matching IEHO on only two. Table 9 demonstrates that after the removal of any one of the corresponding strategies from IEHO, the rank mean values of the resulting variants are all larger than that of the original IEHO, confirming that all three proposed strategies affect the performance of IEHO. Notably, amongst the three improvement strategies, the improved mother–son breeding phase described in Section 3.2.2 has the greatest impact on the IEHO algorithm.

### 4.3. Performance Comparison Between IEHO and the Existing Optimiser

For a comprehensive assessment of the IEHO’s optimisation capability, this section tests it on the CEC2017 benchmark suite against the original algorithm and seven other excellent algorithms, including the Improved Crested Porcupine Optimiser (ICPO [26]), Hybrid Improved Crested Porcupine Optimiser (HICPO) [27], Improved Snake Optimiser (ISO) [28], Enhanced Elk Herd Optimiser (EQDEHO), Improved Genghis Khan Shark Optimiser (IGKSO) [29], Modified Genghis Khan Shark Optimiser (MGKSO) [30], and Improved Circulatory System-Based Optimiser (ICSBO) [31]. For equitable comparison, all competing algorithms are uniformly configured with a population size (N) of 50, a dimensionality (D) of 30, and an allocation of 100,000 function evaluations (denoted as max_FE). IEHO’s remaining hyperparameters are identical to those detailed in Section 3.1 and Section 3.2, and the parameter values of other comparison algorithms are consistent with those specified in their original references, as specifically shown in Table 10.

#### 4.3.1. Algorithm Convergence Accuracy Comparison Experiment

Table 11, Table 12 and Table 13 present the statistical results of the IEHO algorithm and other algorithms on the CEC2017 test suite, with dimensions of 30, 50, and 100, respectively, after 30 independent runs. The boldfaced data indicate the best results achieved on each test function. To further examine the statistically significant differences between the IEHO algorithm and other algorithms, this study employs the Wilcoxon rank-sum test and the Friedman test with a significance level of 5%. The specific results are shown in Table 14, Table 15, Table 16 and Table 17, where Table 13, Table 14, Table 15 and Table 16 present the Wilcoxon rank-sum test results between IEHO and the other improved algorithms at dimensions of 30, 50 and 100, respectively. Table 17 lists the Friedman test results of all algorithms at dimensions of 30, 50 and 100.

From the statistical results in Table 11, amongst the 29 test functions of the 30-dimensional CEC2017 test suite, IEHO achieves the global optimum on 19 functions, namely, F3, F4, F5, F7, F8, F12, F13, F16, F17, F18, F20, F21, F22, F23, F24, F25, F26, F28 and F29. EHO obtains the global optimum on F27; IGKSO achieves the global optimum on F10; and ICSBO attains the global optimum on nine functions, specifically F1, F6, F9, F11, F14, F15, F19, F22 and F30. The remaining comparison algorithms fail to achieve the global optimum on any function. In terms of the number of global optima obtained, IEHO exhibits the highest convergence accuracy. According to the meanings of the ‘+’, ‘−’ and ‘=’ symbols in the Wilcoxon rank-sum test presented in Table 14, EQDEHO, EHO and HICPO do not surpass IEHO on any test function. By contrast, ICPO, ISO, IGKSO and ICSBO outperform IEHO on 2, 1, 1 and 9 functions, respectively. With regard to ties, EQDEHO, EHO, ICPO, ISO, IGKSO, MGKSO, ICSBO and HICPO tie with IEHO on 1, 2, 2, 1, 2, 1, 8 and 1 function(s), respectively, but perform worse on 28, 27, 25, 27, 27, 28, 12 and 28 functions, respectively. These results highlight the robust performance of IEHO on the majority of test functions. Overall, at the test dimension of 30, IEHO demonstrates slightly better robustness than ICSBO and significantly better robustness than all other algorithms. Finally, based on the Friedman results in Table 17, IEHO has the smallest mean rank, ranking first amongst all compared algorithms.

In Table 12, the statistical results demonstrate that amongst the 29 test functions of the 50-dimensional CEC2017 test suite, IEHO achieves the global optimum on 14 functions, namely, F4, F5, F7, F8, F12, F13, F16, F17, F18, F20, F21, F22, F23 and F25. EHO reaches the global optimum on F28; IGKSO achieves the global optimum on F10; and ICSBO attains the global optimum on 11 functions, specifically F1, F3, F6, F9, F11, F14, F15, F19, F24, F26, and F27. The remaining comparison algorithms fail to achieve the global optimum on any function. In terms of the number of global optima obtained by each algorithm, IEHO outperforms the other algorithms in overall effectiveness. According to the meanings of the ‘+’, ‘−’, and ‘=’ symbols in the Wilcoxon rank-sum test presented in Table 15, MGKSO fails to surpass IEHO on any test function and is comparable to IEHO on only one function. HICPO outperforms IEHO on only one function and performs worse than IEHO on all other functions. EQDEHO, EHO, ICPO, ISO, IGKSO and ICSBO outperform IEHO on 2, 3, 3, 1, 1 and 10 functions, respectively; they tie with IEHO on 1, 2, 4, 2, 5 and 8 functions, respectively; but they perform worse on 26, 24, 22, 26, 23 and 11 functions, respectively. In general, at the test dimension of 50, IEHO exhibits slightly better performance than ICSBO and demonstrates consistently superior or competitive performance on the majority of test functions compared with all other algorithms. According to the Friedman results in Table 17, IEHO has the smallest mean rank, indicating that IEHO achieves the best overall performance.

From the results in Table 13, amongst the 29 test functions of the 100-dimensional CEC2017 test suite, IEHO achieves the global optimum on 14 test functions, namely, F4, F7, F8, F12–F19, F21, F25 and F29. EHO realises the global optimum on F27 and F28; ICPO achieves the global optimum only on F20; ISO reaches the global optimum on F22; IGKSO achieves the global optimum on F10; and ICSBO attains the global optimum on 10 test functions, specifically F1, F3, F5, F6, F9, F11, F23, F24, F26 and F30. The remaining comparison algorithms fail to achieve the global optimum on any function. With respect to the number of global optima obtained, IEHO exhibits the highest convergence accuracy. According to the meanings of the ‘+’, ‘−’ and ‘=’ symbols in the Wilcoxon rank-sum test presented in Table 16, HICPO outperforms IEHO on only one function, ties with IEHO on one function, and performs worse than IEHO on all other functions. MGKSO ties with IEHO on only one function and performs worse on all remaining functions. EQDEHO, EHO, ICPO, ISO, IGKSO, and ICSBO outperform IEHO on 1, 2, 2, 2, 4, and 9 functions, respectively; they tie with IEHO on 2, 1, 3, 2, 2, and 9 functions, respectively; and they perform worse on 26, 26, 24, 25, 23, and 11 functions, respectively. Overall, at the test dimension of 100, IEHO exhibits slightly better performance than ICSBO and consistently superior performance on the majority of test functions compared with all other algorithms. The Friedman results in Table 17 demonstrate that IEHO has the smallest mean rank, indicating that IEHO achieves the best performance. In summary, the proposed IEHO algorithm offers certain advantages in convergence accuracy.

#### 4.3.2. Algorithm Convergence Speed Comparison Experiment

To evaluate the convergence speed of the proposed IEHO algorithm, we conducted a comparative analysis based on the running time required by each algorithm to achieve the same number of iterations and the same solution accuracy. The experimental results are summarised in Table 18 and Table 19, where the shorter running times are highlighted in bold. All experiments were conducted under the condition that the test function dimensionality was set to 30 and the maximum number of function evaluations was fixed at max_FE = 100,000. The parameter configurations of IEHO, unless otherwise specified, were consistent with those described in Section 3.1 and Section 3.2, while the parameters of the other algorithms were configured according to Table 10.

As shown in Table 18, when reaching the same number of iterations, the IEHO algorithm does not outperform any of the other algorithms in total runtime across all test functions, indicating that its overall computational speed is relatively slow. The reason is that, as described in Section 3.1, the IEHO algorithm incorporates multiple improvement strategies that significantly increase its computational complexity whilst introducing additional computational operations during the search for superior individuals. Nevertheless, Table 19 emphasises that when achieving the same solution accuracy, the IEHO algorithm converges to the same precision in a shorter time than other algorithms on 13 out of all 29 test functions. In comparison, EQDEHO, ICPO, ISO, IGKSO, ICSBO and HICPO exhibit faster convergence speeds on only 3, 4, 1, 2, 5 and 1 function(s), respectively, whilst neither EHO nor MGKSO demonstrates any time advantage on any function. These results clearly indicate that, despite the longer per-iteration runtime, the IEHO algorithm achieves the best convergence efficiency amongst all compared algorithms. This advantage is attributed to the effective improvement strategies introduced in Section 3.1, which are capable of more accurately guiding the population towards the global optimum and significantly reducing ineffective searches. Consequently, the overall time required to reach the target accuracy is substantially shortened, which fully verifies the superiority of the IEHO algorithm in terms of solution efficiency over other comparison algorithms.

**Figure 5:**
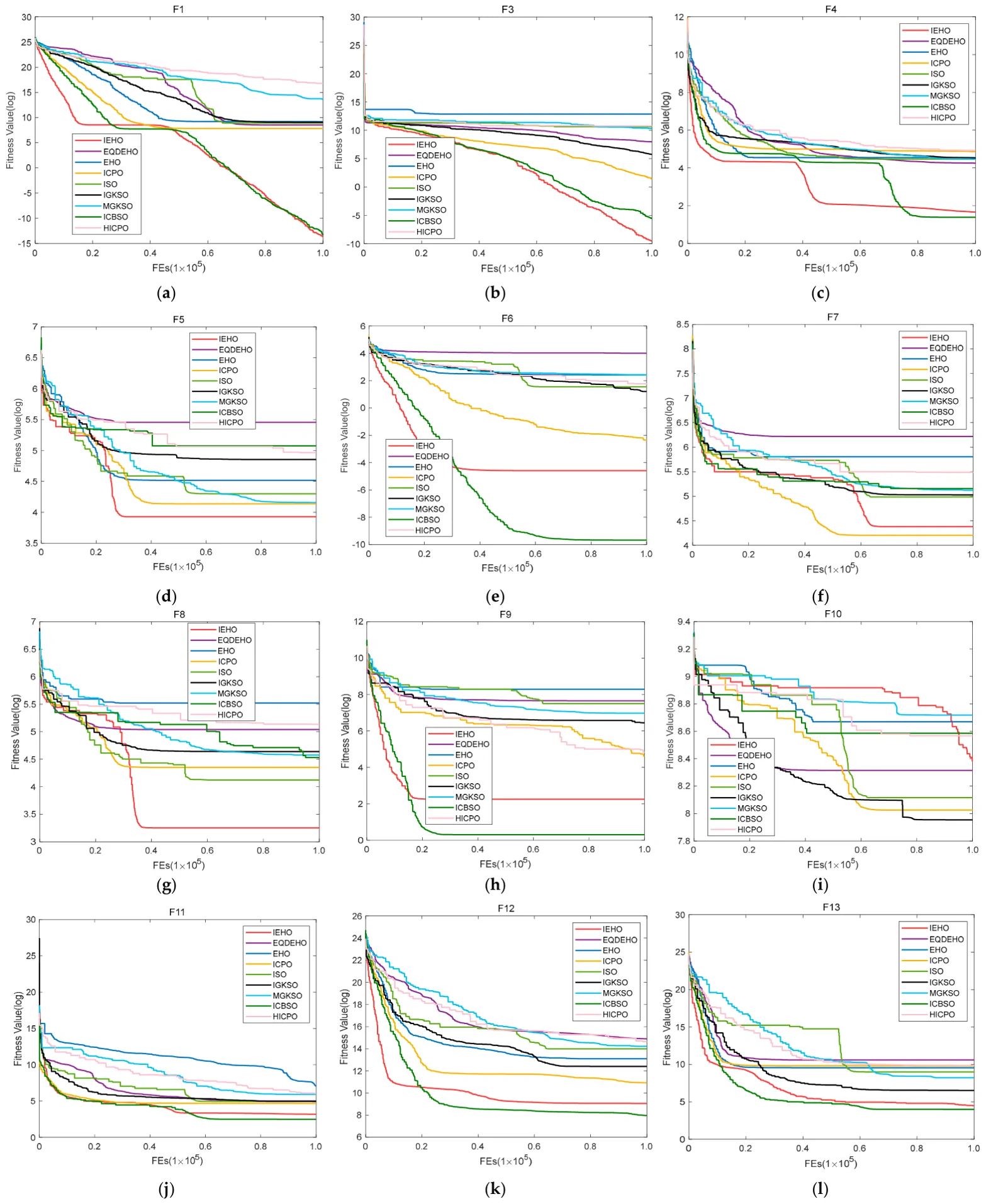

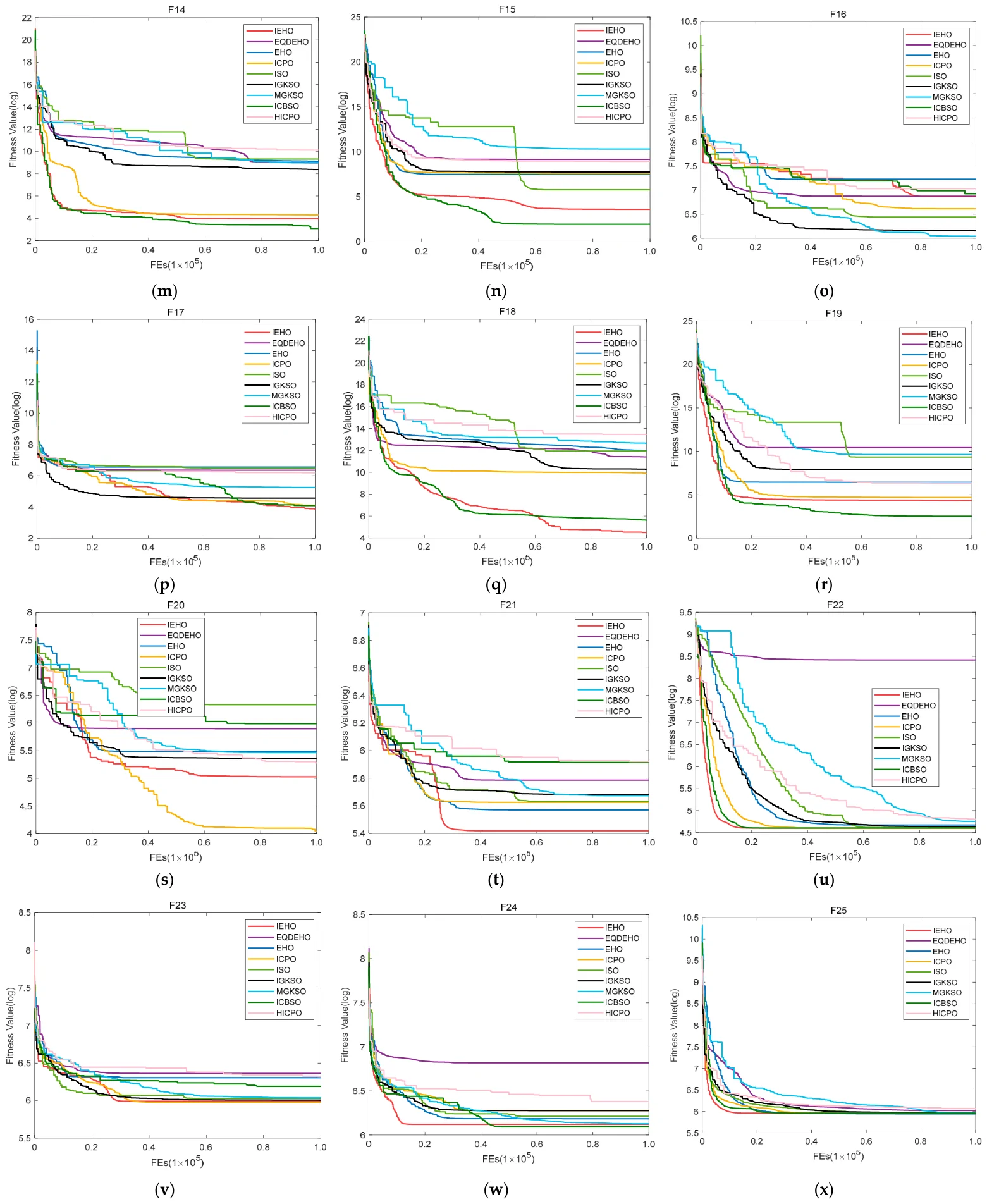

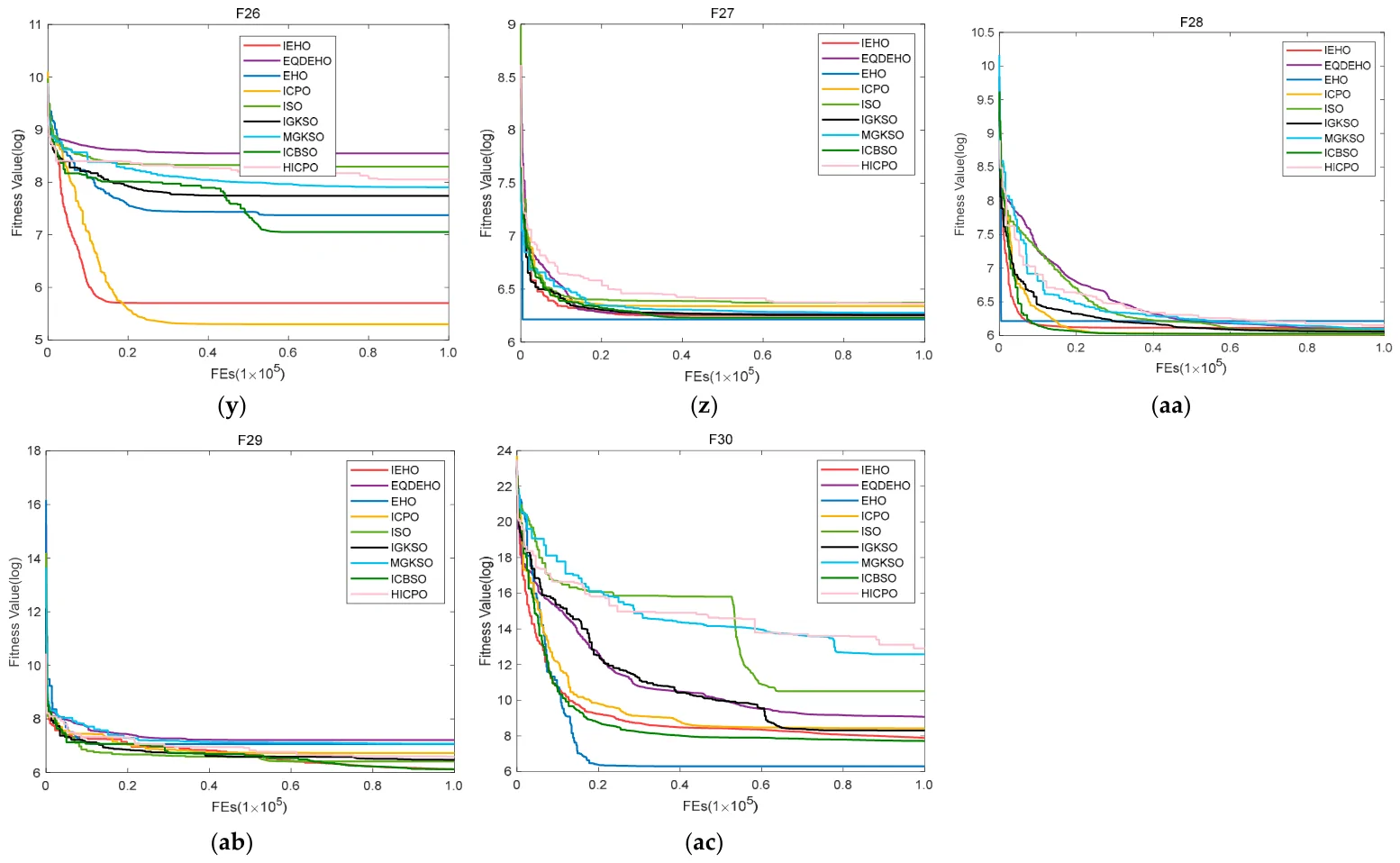
Convergence-curves of each algorithm on CEC2017 test functions.

To further objectively evaluate the convergence performance of IEHO, each subfigure in Figure 5 is analysed based on function type. Unimodal functions contain only a single optimal solution, thus imposing high requirements on the convergence speed of the algorithm. On such problems, IEHO enables the stags to be more uniformly distributed across the search space during the improved rutting stage, thereby covering a broader search region with fewer iterations. Meanwhile, during the improved stag evolution stage (Section 3.2.1), the stags further perform local exploitation within their respective regions. These two improvements jointly ensure that the algorithm can rapidly lock onto the optimal region and maintain continuous convergence on unimodal functions. Consequently, for unimodal functions F1 and F3, IEHO maintains the fastest convergence speed from the early stage of iteration and ultimately achieves the best convergence accuracy.

Multimodal functions F4−F10 possess multiple local optima, requiring the algorithm to not only quickly locate promising regions but also possess the ability to escape from local optima. To meet this requirement, IEHO adopts the maximin Euclidean distance criterion to determine the stags during the rutting stage and assigns the k nearest individuals to each stag to form a family, ensuring that the families are relatively dispersed across the feasible region. This allows simultaneous local exploitation across multiple search intervals, effectively enhancing population diversity and preventing premature convergence. Meanwhile, during the search process of different families, the hinds adopt an adaptive step size based on the truncated Cauchy distribution. This step size automatically increases in later iterations when the differences among individuals diminish and population diversity declines, thereby enhancing the ability to escape local optima. Benefiting from the above mechanisms, IEHO achieves both the fastest convergence speed and the highest final accuracy on F4, F5, and F8. On F6, F7, and F9, although ICSBO achieves slightly better final accuracy, IEHO exhibits significantly faster convergence speed. On F10, however, the final accuracy of IEHO is inferior to that of IGKSO, EQDEHO, ISO, and ICPO.

Hybrid functions and composite functions F11−F30 contain a large number of irregular local traps, requiring the algorithm not only to locate the optimal region but also to possess the ability to escape from complex local traps. To this end, IEHO enables families to search in parallel across different subregions during the improved rutting stage, broadening the search scope. After the stag evolution is completed, an opposition-based learning strategy is introduced to increase the probability of escaping local optima. During the hind evolution, a dimensional crossover strategy with stags is employed to accelerate convergence. For free individuals far from the stags, different tasks are assigned according to their quality−high-quality free individuals perform refined local exploitation within the discovered promising regions, while low−quality free individuals, guided by the grouped individuals, conduct extensive global exploration across the entire feasible region to continuously supplement population diversity. In addition, the step−size factor based on the truncated Cauchy distribution adaptively adjusts according to changes in population diversity, generating larger perturbation steps when diversity declines to assist individuals in escaping local traps. Through the synergistic coordination of the above multiple strategies, IEHO demonstrates satisfactory overall performance on complex functions such as F11−F30. Specifically, on F11−F15 and F19, IEHO achieves the fastest convergence speed in the early stage, with final accuracy reaching a suboptimal level. On F16, although the final accuracy is somewhat insufficient, the early convergence speed is the fastest among all compared algorithms. On F17, the convergence speed is slightly slower than that of some compared algorithms, but the final accuracy is the best. On F18, the initial convergence is relatively slow, but the speed accelerates in the middle and later stages, ultimately achieving the optimal accuracy. On F20 and F26, IEHO achieves the fastest convergence speed, but its final accuracy is inferior to that of ICPO. On F21, F22, F23, and F25, both the convergence speed and final accuracy are optimal. On F24, the convergence speed is the fastest, while the final accuracy is slightly inferior to that of ICSBO. On F27 and F28, the differences in final accuracy are relatively small, and the convergence speed remains competitive. On F30, IEHO achieves comparable accuracy to ICSBO, but both are inferior to EHO. Overall, IEHO exhibits favourable convergence performance on the majority of hybrid and composite functions.

## 5. Conclusions

This paper proposes an Improved Elk Herd Optimiser (IEHO) to further enhance the convergence performance of the original algorithm and mitigate its shortcomings of insufficient diversity and susceptibility to local optima. First, in the rutting phase, the number of stags is reduced, and a novel family grouping strategy is proposed, where stags are determined by considering both fitness and distance, and the N individuals closest to each stag are grouped into a family. This ensures that families are relatively evenly distributed across different regions of the search space, thereby maintaining population distribution and alleviating the loss of population diversity. In the calving phase, new individual update strategies and adaptive step-size factors are proposed. During the stag evolution stage, communication with other dominant individuals is introduced to increase evolutionary directions, and the degree of learning from other individuals is adjusted according to the iteration number. After the evolution is completed, an opposition-based learning strategy is introduced to further enhance the algorithm’s ability to escape local optima. During the evolution of females and free individuals, the Cauchy step-size factor is employed to increase evolutionary directions, while population diversity is used to control the step size, and a dimensional crossover strategy is introduced to enhance population diversity. In addition, in the selection phase, a one-to-one elite retention method between new individuals and original individuals is adopted to determine the iterative individuals, which avoids the decline in population diversity caused by excessively rapid convergence in the original algorithm. Meanwhile, experimental results on the CEC2017 test suite with dimensions of 30, 50, and 100 demonstrate that the proposed IEHO algorithm achieves the best mean results on 18, 14, and 14 out of 29 test functions, respectively. These quantitative results indicate that IEHO has a significant advantage over other competing algorithms in terms of the number of functions on which it achieves the optimal value.

## Figures and Tables

**Table 1:** Effect of parameter Br on IEHO.

Br						
Function		0.04	0.1	0.2	0.3	0.4
F1	Mean	3.25E−01	**2.46E−06**	2.56E−06	2.97E−06	4.26E−06
	Std	1.05E+00	**1.17E−06**	7.39E−06	8.27E−06	1.04E−05
F3	Mean	**8.72E−11**	1.03E−07	1.07E−02	1.33E+03	9.72E+03
	Std	**1.80E−10**	2.40E−07	2.54E−02	8.95E+02	3.22E+03
F4	Mean	2.57E+01	7.95E+00	1.89E+01	**5.52E+00**	6.02E+00
	Std	3.04E+01	1.43E+01	2.47E+01	**1.24E+01**	7.37E+00
F5	Mean	5.07E+01	**4.49E+01**	5.27E+01	5.72E+01	6.45E+01
	Std	1.09E+01	**1.05E+01**	1.51E+01	9.62E+00	4.11E+01
F6	Mean	5.03E−01	7.62E−02	7.54E−02	1.85E−02	**4.45E−03**
	Std	6.10E−01	1.20E−01	9.27E−02	3.66E−02	**1.58E−02**
F7	Mean	8.18E+01	**7.62E+01**	7.92E+01	8.86E+01	1.75E+02
	Std	1.34E+01	**1.34E+01**	1.23E+01	4.45E+01	3.67E+01
F8	Mean	5.35E+01	**4.16E+01**	4.80E+01	5.10E+01	6.89E+01
	Std	1.42E+01	**1.28E+01**	1.29E+01	1.26E+01	4.30E+01
F9	Mean	3.06E+01	1.25E+01	1.21E+01	5.51E+00	**1.59E+00**
	Std	3.67E+01	2.20E+01	1.69E+01	6.11E+00	**1.60E+00**
F10	Mean	**3.34E+03**	4.78E+03	3.82E+03	6.18E+03	6.73E+03
	Std	**1.40E+03**	1.85E+03	1.66E+03	6.88E+02	4.55E+02
F11	Mean	1.24E+02	3.93E+01	3.68E+01	**2.06E+01**	3.12E+01
	Std	1.09E+02	2.20E+01	1.76E+01	**1.36E+01**	3.00E+01
F12	Mean	9.54E+03	**6.84E+03**	6.90E+03	9.30E+03	1.18E+04
	Std	6.81E+03	**5.43E+03**	4.37E+03	4.30E+03	5.58E+03
F13	Mean	2.33E+02	**1.16E+02**	1.76E+02	8.29E+02	1.53E+02
	Std	3.80E+02	**5.17E+01**	3.85E+02	3.70E+03	3.87E+01
F14	Mean	8.55E+01	**5.54E+01**	6.92E+01	5.24E+01	6.79E+01
	Std	2.50E+01	**1.27E+01**	1.24E+01	1.35E+01	9.18E+00
F15	Mean	1.59E+02	5.63E+01	5.57E+01	**3.01E+01**	4.84E+01
	Std	7.85E+01	3.94E+01	3.25E+01	**1.81E+01**	1.74E+01
F16	Mean	4.97E+02	**4.02E+02**	4.21E+02	8.95E+02	1.30E+03
	Std	3.18E+02	**7.18E+01**	2.68E+02	5.01E+02	2.37E+02
F17	Mean	1.30E+02	**6.38E+01**	6.82E+01	7.19E+01	1.18E+02
	Std	7.55E+01	**4.32E+01**	5.19E+01	2.67E+01	6.26E+01
F18	Mean	1.20E+02	**8.72E+01**	1.20E+02	8.99E+01	9.08E+01
	Std	1.15E+02	**9.69E+01**	1.32E+02	1.34E+01	1.67E+01
F19	Mean	7.65E+01	3.92E+01	4.22E+01	2.33E+01	**2.99E+01**
	Std	3.49E+01	1.97E+01	3.08E+01	5.08E+00	**3.02E+00**
F20	Mean	1.68E+02	**9.37E+01**	1.29E+02	1.05E+02	1.81E+02
	Std	8.75E+01	**6.10E+01**	6.59E+01	8.09E+01	8.36E+01
F21	Mean	2.49E+02	**2.38E+02**	2.46E+02	2.66E+02	2.52E+02
	Std	1.11E+01	**1.19E+01**	9.25E+00	1.11E+01	4.17E+01
F22	Mean	1.01E+02	1.00E+02	1.00E+02	1.00E+02	1.00E+02
	Std	1.38E+00	7.51E−01	8.52E−01	5.99E−01	9.13E−08
F23	Mean	4.09E+02	**3.88E+02**	3.97E+02	3.98E+02	3.94+02
	Std	1.91E+01	**1.41E+01**	1.32E+01	1.11E+01	1.51E+01
F24	Mean	4.91E+02	4.61E+02	4.60E+02	4.56E+02	**4.53E+02**
	Std	2.80E+01	1.63E+01	1.63E+01	1.02E+01	**1.47E+01**
F25	Mean	3.96E+02	3.88E+02	3.90E+02	3.87E+02	**3.87E+02**
	Std	1.63E+01	3.77E+00	8.96E+00	1.12E+00	**6.91E−01**
F26	Mean	1.27E+03	1.29E+03	1.23E+03	8.41E+02	**6.83E+02**
	Std	8.84E+02	5.04E+02	6.49E+02	5.94E+02	**5.49E+02**
F27	Mean	5.32E+02	**5.18E+02**	5.29E+02	5.72E+02	6.10E+02
	Std	1.38E+01	**1.32E+01**	1.14E+01	7.82E+00	7.18E+00
F28	Mean	3.36E+02	**3.20E+02**	3.33E+02	3.30E+02	3.28E+02
	Std	5.79E+01	**4.73E+01**	5.22E+01	5.57E+01	4.64E+01
F29	Mean	5.89E+02	5.04E+02	5.20E+02	**5.00E+02**	5.85E+02
	Std	7.81E+01	5.24E+01	7.92E+01	**5.76E+01**	1.17E+02
F30	Mean	3.55E+03	**3.03E+03**	3.43E+03	3.61E+03	5.05E+03
	Std	1.67E+03	**5.57E+02**	1.11E+03	5.76E+02	1.37E+03
Avg.rank sort		4.09	2.03	2.82	2.76	3.34
		5	1	3	2	4

**Table 2:** Effect of parameter k on IEHO.

k						
Function		5	6	7	8	9
F1	Mean	5.45E−05	**4.50E−07**	2.46E−06	4.26E−04	2.43E−05
	Std	2.88E−04	**9.44E−07**	1.17E−06	1.04E−05	7.42E−06
F3	Mean	5.04E−07	1.04E−06	**1.03E−07**	9.72E+03	1.42E−07
	Std	1.88E−06	2.40E−06	**2.40E−07**	3.22E+03	2.35E−07
F4	Mean	6.40E+00	8.95E+00	7.95E+00	**6.02E+00**	1.61E+01
	Std	1.26E+01	1.44E+01	1.43E+01	**7.37E+00**	2.49E+01
F5	Mean	4.80E+01	4.97E+01	**4.49E+01**	6.45E+01	4.81E+01
	Std	1.12E+01	1.05E+01	**1.05E+01**	4.11E+01	1.13E+01
F6	Mean	7.42E−02	7.60E−02	7.62E−02	**4.45E−03**	3.53E−02
	Std	1.26E−01	1.20E−01	1.20E−01	**1.58E−02**	5.39E−02
F7	Mean	7.27E+01	7.69E+01	7.62E+01	1.75E+02	**6.85E+01**
	Std	1.02E+01	1.37E+01	1.34E+01	3.67E+01	**1.23E+01**
F8	Mean	4.18E+01	5.17E+01	**4.16E+01**	6.89E+01	5.93E+01
	Std	1.19E+01	1.28E+01	**1.28E+01**	4.30E+01	1.23E+01
F9	Mean	7.37E+00	1.26E+01	1.25E+01	1.59E+00	**7.19E+00**
	Std	7.35E+00	2.04E+01	2.20E+01	1.60E+00	**6.44E+00**
F10	Mean	4.91E+03	4.98E+03	**4.78E+03**	6.73E+03	5.15E+03
	Std	1.73E+03	1.85E+03	**1.85E+03**	4.55E+02	1.65E+03
F11	Mean	3.64E+01	4.03E+01	3.93E+01	**3.12E+01**	3.23E+01
	Std	1.92E+01	2.21E+01	2.20E+01	**3.00E+01**	1.38E+01
F12	Mean	9.80E+03	6.85E+03	**6.84E+03**	1.18E+04	7.42E+03
	Std	5.07E+03	5.44E+03	**5.43E+03**	5.58E+03	5.09E+03
F13	Mean	1.67E+02	1.19E+02	**1.16E+02**	1.53E+02	1.31E+02
	Std	2.13E+02	5.18E+01	**5.17E+01**	3.87E+01	5.15E+01
F14	Mean	4.96E+01	5.54E+01	5.54E+01	6.79E+01	**5.05E+01**
	Std	1.07E+01	1.27E+01	1.27E+01	9.18E+00	**2.02E+01**
F15	Mean	7.19E+01	5.64E+01	**5.63E+01**	5.84E+01	6.79E+01
	Std	3.90E+01	3.94E+01	**3.94E+01**	1.74E+01	4.12E+01
F16	Mean	4.11E+02	5.01E+02	**4.02E+02**	1.30E+03	4.62E+02
	Std	3.18E+02	2.95E+02	**7.18E+01**	2.37E+02	3.25E+02
F17	Mean	**5.69E+01**	6.39E+01	6.38E+01	1.18E+02	6.11E+01
	Std	**2.99E+01**	4.32E+01	4.32E+01	6.26E+01	4.21E+01
F18	Mean	**6.99E+01**	8.73E+01	8.72E+01	9.08E+01	9.67E+01
	Std	**3.83E+01**	9.69E+01	9.69E+01	1.67E+01	7.49E+01
F19	Mean	4.39E+01	3.93E+01	3.92E+01	**2.99E+01**	4.73E+01
	Std	2.57E+01	1.97E+01	1.97E+01	**3.02E+00**	3.28E+01
F20	Mean	1.21E+02	9.37E+01	9.37E+01	1.81E+02	**9.11E+01**
	Std	6.66E+01	6.10E+01	6.10E+01	8.36E+01	**6.75E+01**
F21	Mean	**2.36E+02**	2.38E+02	2.38E+02	2.52E+02	2.37E+02
	Std	**8.78E+00**	1.12E+01	1.19E+01	4.17E+01	1.04E+01
F22	Mean	1.00E+02	1.00E+02	1.00E+02	1.00E+02	1.00E+02
	Std	8.53E−01	7.52E−01	7.51E−01	9.13E−08	9.45E−01
F23	Mean	3.92E+02	3.89E+02	**3.88E+02**	3.94E+02	3.94E+02
	Std	1.52E+01	1.41E+01	**1.41E+01**	1.51E+01	1.48E+01
F24	Mean	4.88E+02	4.71E+02	**4.61E+02**	4.83E+02	4.67E+02
	Std	1.56E+01	1.63E+01	**1.63E+01**	1.47E+01	1.81E+01
F25	Mean	3.90E+02	3.92E+02	**3.83E+02**	3.87E+02	3.91E+02
	Std	7.29E+00	3.77E+00	**3.77E+00**	6.91E−01	1.34E+01
F26	Mean	1.38E+03	1.29E+03	1.29E+03	**6.83E+02**	1.38E+03
	Std	4.76E+02	5.04E+02	5.04E+02	**5.49E+02**	4.26E+02
F27	Mean	5.20E+02	5.18E+02	5.18E+02	**5.10E+02**	5.20E+02
	Std	1.05E+01	1.38E+01	1.32E+01	**7.18E+00**	1.18E+01
F28	Mean	3.41E+02	3.27E+02	**3.20E+02**	3.28E+02	3.28E+02
	Std	6.12E+01	4.74E+01	**4.73E+01**	4.64E+01	5.83E+01
F29	Mean	5.02E+02	5.14E+02	**5.04E+02**	5.85E+02	4.92E+02
	Std	4.67E+01	5.25E+01	**5.24E+01**	1.17E+02	5.58E+01
F30	Mean	3.19E+03	3.14E+03	**3.03E+03**	5.05E+03	3.33E+03
	Std	8.46E+02	5.57E+02	**5.56E+02**	1.37E+03	1.56E+03
Avg.rank sort		2.89	3.07	**2.20**	3.61	3.2
		2	3	**1**	4	5

**Table 3:** Effect of Pmin and Pmax on IEHO.

Pmin–Pmax						
Function		0–0.4	0.1–0.5	0.2–0.6	0.3–0.7	0.4–0.8
F1	Mean	9.79E−05	**2.46E−06**	5.21E−04	1.12E−05	6.35E−06
	Std	3.48E−05	**1.17E−06**	1.24E−04	3.86E−06	2.65E−06
F3	Mean	4.80E−07	1.03E−07	2.82E−07	6.86E−07	2.73E−07
	Std	1.61E−06	2.40E−07	7.50E−07	1.74E−06	5.24E−07
F4	Mean	1.59E+01	**7.95E+00**	1.38E+01	1.04E+01	1.15E+01
	Std	2.50E+01	**1.43E+01**	2.25E+01	1.84E+01	1.28E+01
F5	Mean	4.95E+01	**4.49E+01**	5.08E+01	4.96E+01	5.98E+01
	Std	1.09E+01	**1.05E+01**	9.01E+00	1.20E+01	1.08E+01
F6	Mean	5.17E−02	7.62E−02	7.14E−02	9.32E−02	3.40E−02
	Std	7.76E−02	1.20E−01	1.12E−01	2.28E−01	5.12E−02
F7	Mean	7.50E+01	7.62E+01	**7.14E+01**	7.37E+01	7.15E+01
	Std	1.27E+01	1.34E+01	**1.44E+01**	1.32E+01	1.32E+01
F8	Mean	4.55E+01	**4.16E+01**	4.17E+01	4.59E+01	4.32E+01
	Std	9.19E+00	**1.28E+01**	1.08E+01	9.03E+00	1.05E+01
F9	Mean	**7.96E+00**	1.25E+01	5.53E+00	8.06E+00	9.62E+00
	Std	**8.77E+00**	2.20E+01	6.42E+00	9.99E+00	1.23E+01
F10	Mean	4.81E+03	4.78E+03	4.99E+03	**3.96E+03**	5.04E+03
	Std	1.93E+03	1.85E+03	1.90E+03	**1.76E+03**	1.76E+03
F11	Mean	4.14E+01	**3.93E+01**	4.54E+01	3.59E+01	4.62E+01
	Std	2.08E+01	**2.20E+01**	3.23E+01	2.07E+01	2.33E+01
F12	Mean	8.42E+03	**6.84E+03**	7.20E+03	8.44E+03	8.07E+03
	Std	5.47E+03	**5.43E+03**	3.69E+03	5.85E+03	5.12E+03
F13	Mean	8.34E+02	**1.16E+02**	2.07E+02	2.20E+02	1.35E+02
	Std	3.94E+03	**5.17E+01**	7.08E+01	5.54E+02	1.11E+02
F14	Mean	**4.78E+01**	5.54E+01	4.99E+01	5.04E+01	4.83E+01
	Std	**1.57E+01**	1.27E+01	1.38E+01	1.56E+01	1.49E+01
F15	Mean	7.23E+01	**5.63E+01**	8.07E+01	6.64E+01	6.38E+01
	Std	6.13E+01	**3.94E+01**	5.20E+01	4.09E+01	3.62E+01
F16	Mean	4.17E+02	**4.02E+02**	4.40E+02	6.20E+02	5.33E+02
	Std	2.79E+02	**7.18E+01**	2.76E+02	4.07E+02	3.43E+02
F17	Mean	5.85E+01	6.38E+01	5.65E+01	**5.45E+01**	6.32E+01
	Std	3.65E+01	4.32E+01	3.49E+01	**2.72E+01**	4.53E+01
F18	Mean	**6.19E+01**	8.72E+01	1.24E+02	1.25E+02	8.91E+01
	Std	**3.57E+01**	9.69E+01	1.21E+02	3.13E+02	1.29E+02
F19	Mean	**3.76E+01**	3.92E+01	4.42E+01	4.29E+01	4.45E+01
	Std	**2.09E+01**	1.97E+01	2.56E+01	2.49E+01	2.38E+01
F20	Mean	1.03E+02	**9.37E+01**	1.04E+02	1.15E+02	1.34E+02
	Std	5.98E+01	**6.10E+01**	6.66E+01	6.60E+01	6.45E+01
F21	Mean	2.77E+02	**2.38E+02**	2.46E+02	2.38E+02	2.50E+02
	Std	9.58E+00	**1.19E+01**	1.02E+01	1.20E+01	1.09E+01
F22	Mean	1.00E+02	1.00E+02	1.00E+02	1.00E+02	1.00E+02
	Std	1.05E+00	7.51E−01	9.54E−01	9.68E−01	6.22E−01
F23	Mean	3.91E+02	**3.88E+02**	3.97E+02	3.93E+02	3.90E+02
	Std	1.38E+01	**1.41E+01**	1.93E+01	1.77E+01	1.43E+01
F24	Mean	4.91E+02	**4.61E+02**	4.75E+02	4.90E+02	4.80E+02
	Std	1.02E+01	**1.63E+01**	1.15E+01	1.56E+01	1.20E+01
F25	Mean	3.88E+02	**3.83E+02**	3.98E+02	3.96E+02	3.90E+02
	Std	3.00E+00	**3.77E+00**	3.32E+00	1.11E+01	8.26E+00
F26	Mean	1.28E+03	1.29E+03	**1.01E+03**	1.00E+03	1.10E+03
	Std	5.40E+02	5.04E+02	**6.68E+02**	6.49E+02	6.06E+02
F27	Mean	5.18E+02	5.18E+02	5.18E+02	5.19E+02	5.19E+02
	Std	1.07E+01	1.32E+01	9.26E+00	1.43E+01	9.95E+00
F28	Mean	3.42E+02	**3.20E+02**	3.38E+02	3.37E+02	3.30E+02
	Std	5.20E+01	**4.73E+01**	5.56E+01	5.49E+01	5.11E+01
F29	Mean	**4.91E+02**	5.04E+02	4.98E+02	5.07E+02	4.99E+02
	Std	**4.50E+01**	5.24E+01	4.77E+01	6.82E+01	5.70E+01
F30	Mean	3.26E+03	**3.03E+03**	3.06E+03	3.19E+03	3.06E+03
	Std	9.99E+02	**5.57E+02**	9.27E+02	8.99E+02	6.56E+02
Avg.rank sort		3.03	**2.10**	3.13	3.44	3.27
		2	**1**	3	5	4

**Table 4:** Effect of g on IEHO.

g						
Function		0.3−0.6	0.4−0.7	0.5−0.8	0.6−0.9	0.7−1
F1	Mean	2.22E−04	6.83E−05	**2.46E−06**	1.01E−05	1.81E−05
	Std	9.15E−04	2.58E−05	**1.17E−06**	5.48E−05	6.09E−05
F3	Mean	5.04E−08	**9.58E−08**	1.03E−07	2.69E−06	4.36E−05
	Std	1.13E−07	**3.20E−07**	2.40E−07	8.94E−06	1.59E−04
F4	Mean	9.64E+00	1.40E+01	**7.95E+00**	2.22E+01	1.98E+01
	Std	1.64E+01	2.17E+01	**1.43E+01**	2.72E+01	2.75E+01
F5	Mean	4.40E+01	4.37E+01	4.49E+01	4.35E+01	**4.02E+01**
	Std	1.35E+01	1.31E+01	1.05E+01	1.51E+01	**1.15E+01**
F6	Mean	1.19E−01	6.06E−02	**7.62E−02**	5.70E−02	4.95E−02
	Std	1.46E−01	8.54E−02	**1.20E−01**	7.84E−02	1.04E−01
F7	Mean	7.60E+01	7.05E+01	7.62E+01	7.26E+01	**6.96E+01**
	Std	1.22E+01	1.03E+01	1.34E+01	1.31E+01	**9.38E+00**
F8	Mean	4.58E+01	4.35E+01	**4.16E+01**	4.77E+01	4.67E+01
	Std	1.15E+01	1.17E+01	**1.28E+01**	9.96E+00	1.14E+01
F9	Mean	1.34E+01	1.61E+01	1.25E+01	7.60E+00	**5.20E+00**
	Std	1.24E+01	1.82E+01	2.20E+01	7.21E+00	**5.86E+00**
F10	Mean	5.47E+03	5.38E+03	**4.78E+03**	5.26E+03	5.97E+03
	Std	1.77E+03	1.67E+03	**1.85E+03**	1.93E+03	1.80E+03
F11	Mean	4.63E+01	5.10E+01	**3.93E+01**	3.97E+01	4.95E+01
	Std	2.67E+01	3.69E+01	**2.20E+01**	2.57E+01	2.99E+01
F12	Mean	9.26E+03	8.89E+03	**6.84E+03**	7.78E+03	7.53E+03
	Std	5.61E+03	5.53E+03	**5.43E+03**	6.75E+03	5.28E+03
F13	Mean	3.99E+02	1.31E+02	**1.16E+02**	1.46E+02	2.46E+02
	Std	7.34E+02	5.68E+01	**5.17E+01**	1.55E+02	7.36E+02
F14	Mean	1.08E+02	7.66E+01	5.54E+01	5.18E+01	**4.32E+01**
	Std	2.82E+01	2.58E+01	1.27E+01	1.86E+01	**1.05E+01**
F15	Mean	1.58E+02	9.95E+01	5.63E+01	5.98E+01	**4.88E+01**
	Std	7.89E+01	4.45E+01	3.94E+01	3.65E+01	**2.96E+01**
F16	Mean	4.09E+02	4.17E+02	**4.02E+02**	4.34E+02	5.03E+02
	Std	3.09E+02	2.29E+02	**7.18E+01**	3.88E+02	3.55E+02
F17	Mean	7.91E+01	7.83E+01	6.38E+01	**5.40E+01**	6.24E+01
	Std	4.95E+01	5.26E+01	4.32E+01	**3.37E+01**	4.83E+01
F18	Mean	3.24E+02	1.53E+02	8.72E+01	8.36E+01	**6.22E+01**
	Std	5.09E+02	1.69E+02	9.69E+01	6.63E+01	**5.11E+01**
F19	Mean	7.74E+01	7.31E+01	**3.92E+01**	4.27E+01	4.72E+01
	Std	2.97E+01	2.97E+01	**1.97E+01**	2.14E+01	1.95E+01
F20	Mean	1.35E+02	9.97E+01	**9.37E+01**	1.31E+02	1.20E+02
	Std	8.27E+01	6.61E+01	**6.10E+01**	8.19E+01	5.71E+01
F21	Mean	**2.41E+02**	2.38E+02	2.38E+02	2.35E+02	2.36E+02
	Std	**1.12E+01**	1.12E+01	1.19E+01	1.20E+01	9.53E+00
F22	Mean	1.01E+02	1.00E+02	1.00E+02	1.00E+02	1.00E+02
	Std	1.15E+00	9.40E−01	7.51E−01	8.53E−01	7.53E−01
F23	Mean	3.97E+02	3.92E+02	**3.88E+02**	3.92E+02	3.89E+02
	Std	1.74E+01	1.38E+01	**1.41E+01**	1.26E+01	1.46E+01
F24	Mean	4.58E+02	4.68E+02	4.61E+02	4.57E+02	**4.54E+02**
	Std	1.32E+01	1.40E+01	1.63E+01	1.26E+01	**1.24E+01**
F25	Mean	3.89E+02	3.90E+02	3.83E+02	3.88E+02	3.87E+02
	Std	5.19E+00	5.87E+00	3.77E+00	2.89E+00	2.45E+00
F26	Mean	1.02E+03	1.11E+03	1.29E+03	**9.40E+02**	1.22E+03
	Std	7.24E+02	5.97E+02	5.04E+02	**6.13E+02**	5.72E+02
F27	Mean	5.25E+02	5.22E+02	5.18E+02	5.16E+02	**5.15E+02**
	Std	1.67E+01	1.68E+01	1.32E+01	8.80E+00	**9.05E+00**
F28	Mean	3.21E+02	3.23E+02	**3.20E+02**	3.25E+02	3.48E+02
	Std	4.30E+01	4.82E+01	**4.73E+01**	4.65E+01	6.17E+01
F29	Mean	5.28E+02	5.11E+02	**5.04E+02**	5.13E+02	5.29E+02
	Std	6.12E+01	5.96E+01	**5.24E+01**	4.56E+01	5.08E+01
F30	Mean	3.06E+03	2.91E+03	3.03E+03	**2.81E+03**	2.93E+03
	Std	9.76E+02	7.22E+02	5.56E+02	**4.69E+02**	5.45E+02
Avg.rank sort		4.17	3.34	2.48	2.83	2.67
		5	4	**1**	3	2

**Table 5:** Effect of h on IEHO.

h						
Function		0.3–0.1	0.4–0.2	0.5–0.3	0.6–0.4	0.7–0.5
F1	Mean	1.91E−04	1.83E−05	**2.46E−06**	4.85E−05	4.81E−05
	Std	4.19E−04	5.63E−06	**1.17E−06**	1.98E−04	1.99E−04
F3	Mean	2.01E−03	4.45E−05	1.03E−07	**5.89E−09**	6.89E−08
	Std	5.50E−03	1.31E−04	2.40E−07	**2.07E−08**	1.69E−07
F4	Mean	1.83E+01	1.65E+01	**7.95E+00**	1.65E+01	1.35E+01
	Std	2.63E+01	2.49E+01	**1.43E+01**	2.55E+01	2.26E+01
F5	Mean	**3.88E+01**	3.95E+01	4.49E+01	4.04E+01	4.46E+01
	Std	**1.20E+01**	1.09E+01	1.05E+01	1.17E+01	1.09E+01
F6	Mean	2.50E−01	3.71E−01	**7.62E−02**	1.33E−01	1.53E−01
	Std	3.43E−01	5.86E−01	**1.20E−01**	2.58E−01	2.37E−01
F7	Mean	7.05E+01	6.87E+01	7.62E+01	**6.84E+01**	7.14E+01
	Std	1.09E+01	1.27E+01	1.34E+01	**1.00E+01**	1.31E+01
F8	Mean	4.41E+01	4.77E+01	**4.16E+01**	4.36E+01	4.61E+01
	Std	1.06E+01	9.97E+00	**1.28E+01**	1.15E+01	1.22E+01
F9	Mean	**8.05E+00**	7.56E+00	1.25E+01	1.20E+01	1.79E+01
	Std	**9.34E+00**	8.51E+00	2.20E+01	1.93E+01	2.00E+01
F10	Mean	4.64E+03	4.79E+03	4.78E+03	4.76E+03	**4.44E+03**
	Std	1.89E+03	1.76E+03	1.85E+03	1.92E+03	**2.01E+03**
F11	Mean	4.78E+01	4.08E+01	**3.93E+01**	5.01E+01	6.97E+01
	Std	1.96E+01	1.88E+01	**2.20E+01**	2.64E+01	6.08E+01
F12	Mean	9.68E+03	8.03E+03	**6.84E+03**	8.10E+03	7.74E+03
	Std	6.62E+03	5.92E+03	**5.43E+03**	5.14E+03	4.64E+03
F13	Mean	6.64E+02	4.82E+02	**1.16E+02**	1.33E+02	6.46E+02
	Std	1.73E+03	2.04E+03	**5.17E+01**	7.50E+01	2.02E+03
F14	Mean	5.80E+01	7.99E+01	**5.54E+01**	8.28E+01	7.44E+01
	Std	1.10E+01	1.14E+01	**1.27E+01**	2.66E+01	2.66E+01
F15	Mean	**3.82E+01**	5.36E+01	5.63E+01	1.14E+02	1.27E+02
	Std	**2.85E+01**	4.23E+01	3.94E+01	5.30E+01	5.69E+01
F16	Mean	4.44E+02	4.26E+02	4.02E+02	4.24E+02	**3.66E+02**
	Std	3.25E+02	3.86E+02	7.18E+01	3.32E+02	**2.94E+02**
F17	Mean	6.19E+01	6.47E+01	6.38E+01	7.01E+01	**6.08E+01**
	Std	3.73E+01	4.67E+01	4.32E+01	4.88E+01	**2.79E+01**
F18	Mean	9.32E+01	9.66E+01	**8.72E+01**	1.46E+02	2.61E+02
	Std	1.87E+01	1.28E+02	**9.69E+01**	1.26E+02	2.30E+02
F19	Mean	2.17E+01	**1.99E+01**	3.92E+01	5.79E+01	6.45E+01
	Std	8.79E+00	**9.45E+00**	1.97E+01	2.91E+01	3.23E+01
F20	Mean	9.80E+01	1.03E+02	**9.37E+01**	1.04E+02	1.14E+02
	Std	7.21E+01	7.96E+01	**6.10E+01**	6.38E+01	6.35E+01
F21	Mean	2.39E+02	2.40E+02	**2.38E+02**	2.39E+02	2.41E+02
	Std	8.81E+00	1.11E+01	**1.19E+01**	1.24E+01	1.30E+01
F22	Mean	1.00E+02	1.00E+02	1.00E+02	1.00E+02	1.00E+02
	Std	9.99E−01	1.07E+00	7.51E−01	3.53E−13	1.02E+00
F23	Mean	3.90E+02	3.89E+02	**3.88E+02**	3.93E+02	3.91E+02
	Std	1.66E+01	1.67E+01	**1.41E+01**	1.54E+01	1.71E+01
F24	Mean	4.58E+02	4.55E+02	4.61E+02	**4.53E+02**	4.67E+02
	Std	1.41E+01	1.33E+01	1.63E+01	**1.54E+01**	1.45E+01
F25	Mean	3.88E+02	3.87E+02	**3.83E+02**	3.89E+02	3.91E+02
	Std	3.35E+00	2.51E+00	**3.77E+00**	5.28E+00	7.86E+00
F26	Mean	1.36E+03	1.32E+03	**1.29E+03**	1.37E+03	1.31E+03
	Std	5.92E+02	5.63E+02	**5.04E+02**	5.28E+02	6.91E+02
F27	Mean	5.34E+02	5.24E+02	**5.18E+02**	5.22E+02	5.29E+02
	Std	1.12E+01	1.05E+01	**1.32E+01**	1.25E+01	1.35E+01
F28	Mean	3.32E+02	3.30E+02	**3.20E+02**	3.22E+02	3.25E+02
	Std	5.51E+01	5.57E+01	**4.73E+01**	4.57E+01	4.07E+01
F29	Mean	**4.76E+02**	4.97E+02	5.04E+02	5.05E+02	5.40E+02
	Std	**3.19E+01**	6.01E+01	5.24E+01	4.66E+01	9.55E+01
F30	Mean	3.05E+03	3.13E+03	**3.03E+03**	3.10E+03	3.18E+03
	Std	6.18E+02	3.80E+02	**5.56E+02**	7.72E+02	1.00E+03
Avg.rank sort		3.03	2.97	2.10	3.31	3.59
		3	2	**1**	4	5

**Table 6:** Parameter initialization settings for each algorithm.

Algorithms	Related Parameters
IEHO	Br = 0.1
IEHO1	Br = 0.2
IEHO2	Br = 0.1
IEHO3	Br = 0.1

**Table 7:** The running results of all improvement strategies on the 30-dimensional CEC2017 test set are summarised as follows.

Function	IEHO	IEHO1	IEHO2	IEHO3
F1	2.46E−06 ± 1.17E−06	1.37E−09 ± 4.14E−09	2.41E−06 ± 7.85E−06	**6.41E+03 ± 5.22E+03**
F3	1.03E−07 ± 2.40E−07	**4.76E+02 ± 5.78E+02**	**3.20E−02 ± 7.52E−02**	**7.26E−03 ± 9.97E−03**
F4	7.95E+00 ± 1.43E+01	**3.05E+01 ± 3.18E+01**	**2.92E+01 ± 3.01E+01**	**4.99E+01 ± 3.25E+01**
F5	4.49E+01 ± 1.05E+01	**5.37E+01 ± 1.28E+01**	4.29E+01 ± 1.40E+01	**6.88E+01 ± 4.77E+01**
F6	7.62E−02 ± 1.20E−01	**2.13E−01 ± 2.35E−01**	**1.38E−01 ± 2.73E−01**	**8.32E−01 ± 6.28E−01**
F7	7.62E+01 ± 1.34E+01	**7.91E+01 ± 1.38E+01**	7.22E+01 ± 1.11E+01	**1.97E+02 ± 4.37E+01**
F8	4.16E+01 ± 1.28E+01	**5.70E+01 ± 1.71E+01**	**4.54E+01 ± 1.23E+01**	**6.96E+01 ± 4.02E+01**
F9	1.25E+01 ± 2.20E+01	**4.13E+01 ± 4.23E+01**	**1.97E+01 ± 4.16E+01**	**4.80E+01 ± 1.06E+02**
F10	4.78E+03 ± 1.85E+03	3.30E+03 ± 9.47E+02	4.16E+03 ± 1.93E+03	**6.76E+03 ± 3.23E+02**
F11	3.93E+01 ± 2.20E+01	**6.74E+01 ± 6.22E+01**	3.73E+01 ± 1.98E+01	**1.59E+02 ± 7.82E+01**
F12	6.84E+03 ± 5.43E+03	**7.45E+03 ± 5.54E+03**	**8.94E+03 ± 5.51E+03**	**1.32E+04 ± 6.68E+03**
F13	1.16E+02 ± 5.17E+01	**8.44E+03 ± 8.83E+03**	**4.13E+03 ± 4.80E+03**	**3.29E+02 ± 6.46E+02**
F14	5.54E+01 ± 1.27E+01	**1.15E+02 ± 2.76E+01**	5.01E+01 ± 1.77E+01	**7.44E+01 ± 2.05E+01**
F15	5.63E+01 ± 3.94E+01	**4.29E+02 ± 9.36E+02**	**5.89E+01 ± 4.03E+01**	**1.35E+02 ± 5.51E+01**
F16	4.02E+02 ± 7.18E+01	**5.14E+02 ± 2.10E+02**	**4.20E+02 ± 2.80E+02**	**1.21E+03 ± 3.98E+02**
F17	6.38E+01 ± 4.32E+01	**1.28E+02 ± 1.01E+02**	**7.59E+01 ± 4.76E+01**	**1.38E+02 ± 8.51E+01**
F18	8.72E+01 ± 9.69E+01	**1.64E+03 ± 4.18E+03**	**2.71E+02 ± 5.75E+02**	**1.53E+02 ± 1.63E+02**
F19	3.92E+01 ± 1.97E+01	**3.90E+02 ± 1.79E+03**	2.82E+01 ± 1.46E+01	**8.50E+01 ± 3.03E+01**
F20	9.37E+01 ± 6.10E+01	**2.34E+02 ± 1.20E+02**	**1.27E+02 ± 7.08E+01**	**3.16E+02 ± 1.27E+02**
F21	2.38E+02 ± 1.19E+01	**2.49E+02 ± 1.48E+01**	**2.39E+02 ± 1.05E+01**	**2.82E+02 ± 5.48E+01**
F22	1.00E+02 ± 7.51E−01	**1.01E+02 ± 1.24E+00**	**1.01E+02 ± 1.23E+00**	1.00E+02 ± 1.10E+00
F23	3.88E+02 ± 1.41E+01	**3.99E+02 ± 1.83E+01**	3.88E+02 ± 1.71E+01	**4.11E+02 ± 2.06E+01**
F24	4.61E+02 ± 1.63E+01	**4.75E+02 ± 1.89E+01**	**4.64E+02 ± 1.68E+01**	**4.91E+02 ± 2.51E+01**
F25	3.83E+02 ± 3.77E+00	**3.89E+02 ± 7.38E+00**	**3.91E+02 ± 1.12E+01**	**3.96E+02 ± 1.93E+01**
F26	1.29E+03 ± 5.04E+02	7.42E+02 ± 7.52E+02	1.07E+03 ± 6.77E+02	**1.40E+03 ± 8.74E+02**
F27	5.18E+02 ± 1.32E+01	**5.30E+02 ± 1.31E+01**	**5.22E+02 ± 1.52E+01**	**5.36E+02 ± 1.62E+01**
F28	3.20E+02 ± 4.73E+01	3.15E+02 ± 4.04E+01	**3.39E+02 ± 5.19E+01**	**3.74E+02 ± 5.72E+01**
F29	5.04E+02 ± 5.24E+01	**6.05E+02 ± 1.51E+02**	5.04E+02 ± 3.86E+01	**6.27E+02 ± 1.58E+02**
F30	3.03E+03 ± 5.57E+02	**3.31E+03 ± 9.92E+02**	**3.37E+03 ± 8.67E+02**	**3.79E+03 ± 9.17E+02**

**Table 8:** Wilcoxon rank-sum test results of IEHO against each improved strategy on the CEC2017 benchmark suite.

	IEHO1	IEHO2	IEHO3
F1	0.000(+)	0.912(=)	0.000(− )
F3	0.000(−)	0.000(−)	0.000(−)
F4	0.001(−)	0.000(−)	0.000(−)
F5	0.005(−)	0.329(=)	0.015(−)
F6	0.001(−)	0.322(=)	0.000(−)
F7	0.525(=)	0.264(=)	0.000(−)
F8	0.000(−)	0.274(=)	0.000(−)
F9	0.000(−)	0.126(=)	0.000(−)
F10	0.006(+)	0.290(=)	0.000(−)
F11	0.057(=)	0.762(=)	0.000(−)
F12	0.455(=)	0.082(=)	0.000(−)
F13	0.000(−)	0.000(−)	0.000(−)
F14	0.000(−)	0.055(=)	0.000(−)
F15	0.000(−)	0.824(=)	0.000(−)
F16	0.528(=)	0.271(=)	0.000(−)
F17	0.000(−)	0.018(−)	0.000(−)
F18	0.000(−)	0.000(−)	0.006(−)
F19	0.002(−)	0.019(+)	0.000(−)
F20	0.000(−)	0.007(−)	0.000(−)
F21	0.005(−)	0.965(=)	0.000(−)
F22	0.217(=)	0.133(=)	0.386(=)
F23	0.003(−)	0.836(=)	0.000(−)
F24	0.002(−)	0.534(=)	0.000(−)
F25	0.291(=)	0.963(=)	0.025(−)
F26	0.023(+)	0.402(=)	0.079(=)
F27	0.001(−)	0.198(=)	0.000(−)
F28	0.721(=)	0.033(−)	0.000(−)
F29	0.001(−)	0.641(=)	0.000(−)
F30	0.375(=)	0.231(=)	0.000(−)
+/=/−	3/8/18	1/21/7	0/2/27

**Table 9:** Friedman test for each algorithm.

		IEHO	IEHO1	IEHO2	IEHO3
**D = 30**	**Avg.rank**	1.41	2.82	2.13	3.62
	**sort**	1	3	2	4

**Table 10:** Parameter initialization settings for each algorithm.

Algorithms	Related Parameters
EHO	Br = 0.2
ICPO	Tf = 0.5; Alpha = 0.1
ISO	*a =* 2
EQDEHO	F0 = 0.5; SF = 0.5
IGKSO	Pheromone probability = 0.35; m = 1.5
MGKSO	M = 1.5; W1 = 0.1
ICSBO	D *=* 5
HICPO	T = 2; alpha = 0.1; Tf = 0.5

**Table 11:** Numerical results for IEHO against competing optimisers on the CEC2017 benchmark (30-dimensional).

Function		IEHO	EQDEHO	EHO	ICPO	ISO	IGKSO	MGKSO	ICSBO	HICPO
F1	Mean	2.46E−06	3.88E+03	4.19E+03	2.36E+02	4.88E+04	7.56E+03	2.58E+05	7.58E−07	4.26E+03
	Std	1.17E−06	4.34E+03	5.74E+03	8.48E+02	4.63E+04	5.58E+03	3.95E+05	1.58E−07	3.22E+03
F3	Mean	1.03E−07	1.73E+03	2.11E+05	9.83E−02	3.71E+04	1.35E+01	2.36E+04	5.07E−04	2.79E+04
	Std	2.40E−07	1.76E+03	2.46E+05	2.61E−01	5.25E+03	1.22E+01	8.61E+03	1.39E−03	8.21E+03
F4	Mean	7.95E+00	9.60E+01	7.64E+01	3.63E+01	1.04E+02	7.45E+01	1.08E+02	1.41E+01	5.05E+02
	Std	1.43E+01	2.24E+01	2.76E+01	3.82E+01	1.51E+01	2.24E+01	2.91E+01	2.39E+01	1.74E+01
F5	Mean	4.49E+01	2.07E+02	1.23E+02	8.44E+01	7.99E+01	1.07E+02	9.48E+01	1.28E+02	5.63E+02
	Std	1.05E+01	3.09E+01	6.81E+01	2.27E+01	1.82E+01	2.02E+01	2.73E+01	5.33E+01	2.24E+01
F6	Mean	7.62E−02	4.99E+01	1.17E+01	2.96E−01	7.56E+00	2.64E+00	2.03E+01	6.39E−04	6.01E+02
	Std	1.20E−01	6.36E+00	7.39E+00	2.73E−01	4.97E+00	1.03E+00	5.22E+00	8.33E−04	4.57E-01
F7	Mean	7.62E+01	4.52E+02	2.40E+02	1.23E+02	1.38E+02	1.42E+02	2.10E+02	1.51E+02	7.99E+02
	Std	1.34E+01	8.82E+01	8.35E+01	2.22E+01	2.52E+01	2.46E+01	5.09E+01	6.33E+01	1.54E+01
F8	Mean	4.16E+01	1.50E+02	1.40E+02	7.64E+01	7.29E+01	9.99E+01	9.18E+01	1.35E+02	8.64E+02
	Std	1.28E+01	2.14E+01	7.65E+01	1.78E+01	1.55E+01	2.23E+01	2.40E+01	4.81E+01	1.69E+01
F9	Mean	1.25E+01	3.13E+03	2.43E+03	9.07E+01	1.52E+03	6.70E+02	1.12E+03	4.77E−01	9.40E+02
	Std	2.20E+01	6.39E+02	1.34E+03	6.94E+01	8.17E+02	4.29E+02	3.99E+02	5.63E−01	4.92E+01
F10	Mean	4.78E+03	4.04E+03	6.28E+03	3.13E+03	3.09E+03	2.52E+03	5.16E+03	5.01E+03	4.14E+03
	Std	1.85E+03	4.31E+02	1.08E+03	8.55E+02	8.35E+02	4.08E+02	1.58E+03	6.71E+02	6.08E+02
F11	Mean	3.93E+01	1.95E+02	2.03E+05	9.80E+01	1.17E+02	1.05E+02	5.61E+02	2.08E+01	1.17E+03
	Std	2.20E+01	9.88E+02	6.32E+05	3.22E+01	3.24E+01	4.67E+01	3.78E+02	1.88E+01	2.47E+01
F12	Mean	6.84E+03	4.58E+05	8.31E+05	6.83E+04	6.78E+05	6.81E+05	1.30E+06	1.79E+04	3.86E+05
	Std	5.43E+03	4.54E+05	1.84E+06	5.79E+04	6.75E+05	4.22E+05	1.53E+06	1.51E+04	2.30E+05
F13	Mean	1.16E+02	2.26E+04	6.81E+04	9.01E+03	8.00E+03	1.39E+04	2.27E+04	1.23E+02	1.17E+04
	Std	5.17E+01	1.94E+04	2.04E+05	9.75E+03	3.85E+03	1.48E+04	2.28E+04	1.73E+02	1.00E+04
F14	Mean	5.54E+01	2.28E+03	1.16E+05	1.27E+02	1.56E+04	1.09E+03	8.83E+03	2.05E+01	6.22E+03
	Std	1.27E+01	2.57E+03	2.56E+05	7.73E+01	1.43E+04	1.02E+03	6.94E+03	1.85E+01	5.46E+03
F15	Mean	5.63E+01	6.21E+03	5.76E+03	1.03E+03	1.26E+03	4.96E+03	1.15E+04	2.05E+01	7.69E+03
	Std	3.94E+01	8.37E+03	7.19E+03	1.60E+03	1.09E+03	7.61E+03	8.97E+03	1.85E+01	6.53E+03
F16	Mean	4.02E+02	1.24E+03	1.91E+03	7.96E+02	7.72E+02	6.53E+02	9.70E+02	9.37E+02	2.41E+03
	Std	7.18E+01	1.53E+03	1.62E+03	3.04E+02	5.19E+02	3.67E+02	7.52E+02	2.75E+02	2.74E+02
F17	Mean	6.38E+01	7.19E+02	7.68E+02	1.75E+02	3.62E+02	2.00E+02	3.70E+02	1.33E+02	1.82E+03
	Std	4.32E+01	2.71E+02	3.08E+02	1.30E+02	1.87E+02	1.30E+02	1.50E+02	9.71E+01	9.06E+01
F18	Mean	8.72E+01	8.40E+04	1.16E+06	1.56E+04	2.48E+05	3.45E+04	3.72E+05	2.44E+02	2.38E+05
	Std	9.69E+01	6.38E+04	1.88E+06	1.42E+04	2.28E+05	1.51E+04	2.99E+05	3.52E+02	1.48E+05
F19	Mean	3.92E+01	7.17E+03	3.77E+03	1.24E+03	4.40E+03	7.58E+03	1.90E+04	1.21E+01	1.29E+04
	Std	1.97E+01	7.81E+03	3.80E+03	2.01E+03	3.57E+03	7.86E+03	1.70E+04	6.73E+00	1.31E+04
F20	Mean	9.37E+01	6.04E+02	6.89E+02	1.89E+02	3.39E+02	2.24E+02	4.68E+02	1.93E+02	2.18E+03
	Std	6.10E+01	1.91E+02	3.42E+02	1.13E+02	1.29E+02	6.81E+01	1.78E+02	1.12E+02	1.14E+02
F21	Mean	2.38E+02	3.87E+02	3.15E+02	2.73E+02	2.64E+02	2.81E+02	2.81E+02	3.37E+02	2.36E+03
	Std	1.19E+01	4.43E+01	7.53E+01	2.27E+01	1.53E+01	1.75E+01	2.80E+01	4.73E+01	1.49E+01
F22	Mean	1.00E+02	2.59E+03	4.15E+03	1.01E+02	1.02E+02	3.12E+02	1.08E+03	1.00E+02	2.30E+03
	Std	7.51E−01	2.41E+03	3.51E+03	1.38E+00	1.81E+00	8.01E+02	2.22E+03	4.47E−01	1.35E+00
F23	Mean	3.88E+02	7.24E+02	4.85E+02	4.48E+02	4.52E+02	4.58E+02	4.42E+02	4.38E+02	2.71E+03
	Std	1.41E+01	1.44E+02	7.92E+01	2.13E+01	2.27E+01	2.59E+01	1.97E+01	6.19E+01	2.25E+01
F24	Mean	4.61E+02	8.13E+02	5.81E+02	5.25E+02	5.13E+02	5.23E+02	4.96E+02	4.74E+02	2.88E+03
	Std	1.63E+01	1.61E+02	9.45E+01	3.22E+01	2.40E+01	3.03E+01	2.23E+01	7.12E+01	2.38E+01
F25	Mean	3.83E+02	4.06E+02	3.92E+02	3.94E+02	4.02E+02	3.99E+02	4.13E+02	3.87E+02	2.89E+03
	Std	3.77E+00	2.21E+01	1.94E+01	1.34E+01	1.40E+01	1.65E+01	2.31E+01	2.78E−01	1.34E+01
F26	Mean	1.29E+03	4.15E+03	2.40E+03	1.76E+03	2.37E+03	2.05E+03	1.83E+03	1.33E+03	4.06E+03
	Std	5.04E+02	2.16E+03	6.07E+02	9.79E+02	1.63E+03	8.66E+03	6.08E+02	2.76E+02	5.79E+02
F27	Mean	5.18E+02	6.78E+02	5.00E+02	5.44E+02	5.63E+02	5.63E+02	5.42E+02	5.05E+02	3.23E+03
	Std	1.32E+01	1.19E+02	8.09E−05	1.51E+01	1.75E+01	2.21E+01	2.38E+01	7.34E+00	9.99E+00
F28	Mean	3.20E+02	4.22E+02	5.00E+02	3.39E+02	4.43E+02	4.04E+02	4.54E+02	3.32E+02	3.22E+03
	Std	4.73E+01	1.59E+01	6.24E−05	5.01E+01	2.52E+01	1.56E+01	2.75E+01	4.98E+01	1.72E+01
F29	Mean	5.04E+02	1.52E+03	1.16E+03	7.29E+02	8.59E+02	7.07E+02	1.06E+03	5.67E+02	3.55E+03
	Std	5.24E+01	3.59E+02	4.23E+02	1.58E+02	2.02E+02	1.60E+02	1.80E+02	1.48E+02	1.27E+02
F30	Mean	3.03E+03	8.09E+03	1.74E+05	6.08E+03	8.35E+03	9.08E+03	7.16E+04	2.76E+03	1.09E+04
	Std	5.57E+02	3.84E+03	7.17E+05	2.49E+03	4.68E+03	3.55E+03	8.57E+04	7.91E+02	3.69E+03

**Table 12:** Numerical results for IEHO against competing optimisers on the CEC2017 benchmark (50-dimensional).

Function		IEHO	EQDEHO	EHO	ICPO	ISO	IGKSO	MGKSO	ICSBO	HICPO
F1	Mean	2.98E+04	7.10E+03	5.23E+03	3.56E+03	1.96E+06	3.14E+04	8.17E+06	**3.08E+03**	1.25E+07
	Std	2.08E+04	7.23E+03	1.20E+04	4.59E+03	8.27E+05	1.21E+04	5.31E+06	**4.26E+03**	5.27E+06
F3	Mean	1.04E+04	4.46E+04	4.01E+05	3.86E+03	1.35E+05	1.15E+04	1.49E+05	**2.17E+03**	1.23E+05
	Std	3.93E+03	1.22E+04	2.40E+05	2.31E+03	1.40E+04	3.60E+03	3.16E+04	**1.72E+03**	2.16E+04
F4	Mean	**8.11E+01**	1.82E+02	1.58E+02	1.46E+02	1.97E+02	1.43E+02	2.02E+02	8.40E+01	6.11E+02
	Std	**4.07E+01**	6.12E+01	5.09E+01	4.93E+01	2.92E+01	3.42E+01	5.27E+01	5.06E+01	4.40E+01
F5	Mean	**1.12E+02**	3.29E+02	2.82E+02	1.76E+02	1.98E+02	2.38E+02	2.46E+02	1.25E+02	6.48E+02
	Std	**2.33E+01**	3.74E+01	1.27E+02	4.37E+01	3.45E+01	3.05E+01	4.58E+01	1.37E+02	2.86E+01
F6	Mean	1.03E+00	5.73E+01	2.99E+01	4.09E+00	2.24E+01	9.95E+00	3.45E+01	**3.27E−02**	6.08E+02
	Std	6.63E−01	4.83E+00	1.52E+01	2.19E+00	6.45E+00	3.68E+00	8.12E+00	**2.69E−02**	1.70E+00
F7	Mean	**1.73E+02**	9.27E+02	5.42E+02	2.87E+02	3.43E+02	3.40E+02	5.74E+02	3.57E+02	9.21E+02
	Std	**2.79E+01**	8.27E+01	1.97E+02	5.09E+01	5.65E+01	5.70E+01	8.65E+01	8.98E+01	3.64E+01
F8	Mean	**1.03E+02**	3.34E+02	2.65E+02	1.95E+02	1.93E+02	2.27E+02	2.25E+02	2.44E+02	9.47E+02
	Std	**2.08E+01**	4.30E+01	1.30E+02	3.34E+01	2.96E+01	3.25E+01	3.72E+01	1.30E+02	3.01E+01
F9	Mean	2.93E+02	1.01E+04	1.18E+04	1.73E+03	1.02E+04	3.55E+03	5.93E+03	**3.06E+01**	2.22E+03
	Std	7.77E+02	1.37E+03	3.89E+03	9.46E+02	3.89E+03	1.21E+03	2.72E+03	**2.15E+01**	5.56E+02
F10	Mean	9.98E+03	6.94E+03	1.03E+04	6.30E+03	5.69E+03	**5.57E+03**	1.10E+04	1.12E+04	7.43E+03
	Std	3.18E+03	6.49E+02	1.89E+03	1.72E+03	2.00E+03	**6.90E+02**	2.53E+03	6.99E+02	9.45E+02
F11	Mean	2.13E+02	2.26E+02	5.98E+05	2.25E+02	4.70E+02	2.18E+02	7.43E+02	**6.68E+01**	2.48E+03
	Std	1.47E+02	1.10E+02	1.42E+06	3.96E+01	1.08E+02	6.27E+01	2.28E+02	**1.90E+01**	5.30E+02
F12	Mean	**6.05E+04**	5.17E+06	4.58E+06	8.46E+05	4.70E+06	4.62E+06	1.98E+07	1.92E+05	4.39E+06
	Std	**3.45E+04**	3.10E+06	4.48E+06	7.84E+05	2.50E+06	2.15E+06	1.18E+07	9.01E+04	1.84E+06
F13	Mean	**2.44E+03**	1.21E+04	5.47E+03	4.46E+03	1.41E+04	6.26E+03	3.88E+04	2.53E+03	3.81E+03
	Std	**3.49E+03**	1.07E+04	4.63E+03	4.27E+03	6.56E+03	7.17E+03	2.49E+04	3.56E+03	2.35E+03
F14	Mean	1.66E+02	4.41E+04	1.11E+06	5.71E+03	1.62E+05	1.59E+04	1.12E+05	**8.62E+01**	8.26E+04
	Std	5.99E+01	3.21E+04	2.91E+06	6.39E+03	1.27E+05	1.39E+04	9.34E+04	**2.94E+01**	6.56E+04
F15	Mean	2.81E+03	7.70E+03	2.20E+05	5.51E+03	7.74E+03	1.04E+04	1.56E+04	**3.38E+02**	6.29E+03
	Std	3.16E+03	6.78E+03	8.44E+05	5.12E+03	5.76E+03	5.97E+03	7.48E+03	**2.57E+02**	4.19E+03
F16	Mean	**7.99E+02**	2.07E+03	2.60E+03	1.48E+03	1.37E+03	1.37E+03	1.81E+03	1.82E+03	3.05E+03
	Std	**2.93E+02**	3.67E+02	9.34E+02	3.96E+02	3.66E+02	3.43E+02	3.62E+02	8.02E+02	3.48E+02
F17	Mean	**9.38E+02**	1.84E+03	2.09E+03	9.55E+02	9.57E+03	9.53E+03	1.42E+03	1.37E+03	2.75E+03
	Std	**4.31E+02**	3.46E+02	6.02E+02	3.06E+02	2.83E+02	1.74E+02	3.76E+02	3.48E+02	2.51E+02
F18	Mean	**9.40E+03**	3.64E+05	4.44E+06	8.65E+04	1.77E+06	1.48E+05	1.87E+06	2.03E+04	1.38E+06
	Std	**7.25E+03**	1.94E+05	9.30E+06	4.73E+04	1.03E+06	9.79E+04	1.80E+06	1.59E+04	8.71E+05
F19	Mean	1.04E+03	1.81E+04	1.50E+04	1.06E+04	1.70E+04	1.77E+04	1.93E+04	**5.96E+01**	1.42E+04
	Std	2.94E+03	9.89E+03	8.73E+03	5.86E+03	7.43E+03	1.09E+04	1.43E+04	**2.91E+01**	7.96E+03
F20	Mean	**6.22E+02**	1.20E+03	1.86E+03	6.33E+02	8.50E+02	6.36E+02	1.17E+03	1.17E+03	2.73E+03
	Std	**4.96E+02**	2.97E+02	6.91E+02	2.46E+02	3.36E+02	1.51E+02	3.61E+02	2.84E+02	2.66E+02
F21	Mean	**2.92E+02**	6.12E+02	4.45E+02	3.80E+02	3.48E+02	4.04E+02	3.94E+02	4.65E+02	2.45E+03
	Std	**2.52E+01**	7.14E+01	1.36E+02	3.59E+01	2.05E+01	2.81E+01	4.21E+01	1.34E+02	2.99E+01
F22	Mean	**3.92E+03**	8.05E+03	1.22E+04	6.46E+03	3.97E+03	6.64E+03	1.17E+04	5.43E+03	8.74E+03
	Std	**4.87E+03**	8.93E+02	1.85E+03	2.86E+03	3.84E+03	5.53E+02	3.32E+03	5.82E+03	2.35E+03
F23	Mean	**5.45E+02**	1.32E+03	7.64E+02	6.55E+02	6.46E+02	7.02E+02	6.81E+02	5.51E+02	2.89E+03
	Std	**2.72E+01**	1.05E+02	1.54E+02	5.28E+01	3.86E+01	6.05E+01	5.77E+01	1.14E+02	3.77E+01
F24	Mean	6.24E+02	1.39E+03	8.17E+02	7.57E+02	7.11E+02	7.70E+02	6.99E+02	**6.61E+02**	3.06E+03
	Std	3.13E+01	2.61E+02	1.62E+02	5.03E+01	7.37E+01	4.90E+01	3.28E+01	**4.75E+01**	3.78E+01
F25	Mean	**5.29E+02**	6.14E+02	5.38E+02	5.38E+02	6.27E+02	6.01E+02	6.50E+02	5.34E+02	3.13E+03
	Std	**4.17E+01**	2.91E+01	2.20E+02	3.15E+01	1.46E+01	2.04E+01	3.46E+01	3.56E+01	3.29E+01
F26	Mean	3.00E+03	9.22E+03	4.13E+03	4.08E+03	6.33E+03	3.16E+03	3.79E+03	**1.83E+03**	5.39E+03
	Std	6.64E+02	1.97E+03	5.27E+02	1.20E+03	2.51E+03	2.51E+03	1.94E+03	**2.38E+02**	6.03E+02
F27	Mean	7.04E+02	1.35E+03	5.00E+02	9.46E+02	9.55E+02	9.22E+02	9.27E+02	**5.91E+02**	3.51E+03
	Std	6.52E+01	3.89E+02	6.73E−05	1.15E+02	7.34E+01	1.26E+02	1.53E+02	**3.44E+01**	6.51E+01
F28	Mean	5.18E+02	5.74E+02	**5.00E+02**	5.45E+02	6.70E+02	5.52E+02	6.19E+02	5.13E+02	3.45E+03
	Std	3.81E+01	3.75E+01	**5.52E−05**	2.92E+01	4.27E+01	4.52E+01	5.05E+01	2.42E+01	4.00E+01
F29	Mean	**7.48E+02**	2.39E+03	2.38E+03	1.28E+03	1.54E+03	1.27E+03	2.02E+03	7.61E+02	4.02E+03
	Std	**2.58E+02**	4.27E+02	9.40E+02	2.60E+02	3.05E+02	3.07E+02	3.82E+02	2.95E+02	2.99E+02
F30	Mean	**7.77E+05**	1.22E+06	6.42E+06	1.75E+06	1.89E+06	1.10E+06	1.17E+07	8.05E+05	1.81E+06
	Std	**1.09E+05**	3.01E+05	1.49E+07	4.50E+05	6.27E+05	1.49E+05	4.25E+06	7.42E+04	2.28E+05

**Table 13:** Numerical results for IEHO against competing optimisers on the CEC2017 benchmark (100-dimensional).

Function		IEHO	EQDEHO	EHO	ICPO	ISO	IGKSO	MGKSO	ICSBO	HICPO
F1	Mean	2.48E+04	5.99E+08	1.44E+09	3.59E+04	2.71E+04	3.83E+06	4.15E+08	**7.16E+03**	3.03E+09
	Std	1.50E+04	4.46E+08	1.76E+09	1.28E+04	3.13E+04	7.67E+05	1.01E+08	**1.01E+04**	5.89E+08
F3	Mean	2.33E+05	2.37E+05	2.13E+06	1.10E+05	3.14E+05	1.71E+05	4.87E+05	**1.05E+05**	4.16E+05
	Std	3.11E+04	2.69E+04	2.41E+06	1.75E+04	2.23E+04	2.18E+04	5.33E+04	**1.64E+04**	3.44E+04
F4	Mean	**2.87E+02**	7.37E+02	7.06E+02	3.43E+02	6.33E+02	4.77E+02	8.43E+02	2.89E+02	1.37E+03
	Std	**6.53E+01**	1.09E+02	3.68E+02	4.38E+01	7.17E+01	6.33E+01	1.13E+02	5.29E+01	1.00E+02
F5	Mean	3.75E+02	8.16E+02	6.72E+02	5.44E+02	5.93E+02	6.13E+02	7.33E+02	**3.02E+02**	1.07E+03
	Std	4.52E+01	5.48E+01	2.07E+02	6.01E+01	7.30E+01	4.23E+01	6.80E+01	**2.29E+02**	6.27E+01
F6	Mean	1.23E+01	6.40E+01	5.18E+01	2.10E+01	4.06E+01	3.00E+01	5.53E+01	**1.61E+00**	6.27E+02
	Std	3.42E+00	3.41E+00	1.58E+01	5.04E+00	4.74E+00	4.21E+00	4.18E+00	**6.15E−01**	2.59E+00
F7	Mean	**5.66E+02**	2.45E+03	1.70E+03	1.12E+03	1.05E+03	1.20E+03	1.93E+03	7.31E+02	1.62E+03
	Std	**9.56E+01**	1.17E+02	3.97E+02	1.83E+02	1.01E+02	1.34E+02	2.37E+02	3.30E+02	8.74E+01
F8	Mean	**3.65E+02**	9.38E+02	6.99E+02	5.73E+02	6.24E+02	6.52E+02	7.55E+02	3.69E+02	1.36E+03
	Std	**5.97E+01**	7.27E+01	1.96E+02	7.88E+01	5.37E+01	6.02E+01	9.32E+01	3.04E+02	6.98E+01
F9	Mean	9.00E+03	2.18E+04	3.84E+04	2.01E+04	5.24E+04	1.42E+04	2.39E+04	**1.37E+03**	1.36E+04
	Std	5.37E+03	1.19E+03	1.05E+04	7.81E+03	1.04E+04	1.67E+03	6.61E+03	**8.66E+02**	3.18E+03
F10	Mean	2.57E+04	2.52E+04	2.69E+04	2.67E+04	2.25E+04	**1.42E+04**	2.89E+04	2.55E+04	2.04E+04
	Std	6.43E+03	1.86E+03	3.47E+03	3.75E+03	5.76E+03	**9.71E+02**	4.13E+03	9.86E+02	1.79E+03
F11	Mean	1.41E+03	7.12E+04	4.25E+07	1.57E+03	1.41E+05	2.00E+03	1.75E+06	**9.72E+02**	6.44E+05
	Std	4.39E+02	3.59E+04	1.35E+08	4.14E+02	1.73E+04	3.38E+02	7.01E+05	**3.37E+02**	3.50E+05
F12	Mean	**1.90E+06**	8.15E+07	2.96E+08	1.29E+07	8.68E+07	7.51E+07	3.32E+08	2.09E+06	2.18E+08
	Std	**8.13E+05**	4.38E+07	8.66E+08	5.28E+06	3.22E+07	2.15E+07	1.31E+08	9.05E+05	5.39E+07
F13	Mean	**3.53E+03**	3.03E+04	1.04E+04	5.94E+03	6.90E+04	6.16E+03	1.79E+05	3.59E+03	1.13E+04
	Std	**3.22E+03**	1.94E+04	4.84E+03	3.71E+03	8.73E+04	3.65E+03	1.33E+05	3.56E+03	2.89E+03
F14	Mean	**4.40E+04**	7.53E+05	1.65E+06	1.17E+05	1.57E+06	2.41E+05	2.16E+06	4.84E+04	1.67E+06
	Std	**3.06E+04**	2.79E+05	1.71E+06	5.40E+04	9.59E+05	1.02E+05	1.04E+06	6.96E+04	8.94E+05
F15	Mean	**1.81E+03**	8.47E+03	3.65E+03	2.64E+03	1.19E+04	2.94E+03	4.39E+05	1.84E+03	3.50E+03
	Std	**1.85E+03**	7.34E+03	4.03E+03	1.90E+03	8.66E+03	3.69E+03	1.59E+06	2.65E+03	1.97E+03
F16	Mean	**2.63E+03**	4.66E+03	6.00E+03	4.00E+03	3.70E+03	4.05E+03	4.73E+03	4.14E+03	5.86E+03
	Std	**6.07E+02**	6.40E+02	2.32E+03	5.83E+02	5.63E+02	7.52E+02	7.95E+02	2.23E+03	5.32E+02
F17	Mean	**2.28E+03**	4.17E+03	5.58E+03	2.95E+03	2.69E+03	3.17E+03	3.78E+03	4.45E+03	4.82E+03
	Std	**6.96E+02**	6.88E+02	1.20E+03	3.99E+02	4.71E+02	4.43E+02	5.45E+02	8.83E+02	4.43E+02
F18	Mean	**1.81E+05**	1.41E+06	4.56E+06	3.16E+05	2.82E+06	4.68E+05	4.26E+06	2.26E+05	3.11E+06
	Std	**5.40E+04**	9.00E+05	9.30E+06	1.50E+05	9.78E+05	2.03E+05	2.13E+06	1.06E+05	1.12E+06
F19	Mean	**1.28E+03**	5.00E+03	2.26E+03	2.89E+03	1.56E+03	1.37E+03	2.68E+05	1.43E+03	3.84E+03
	Std	**1.56E+03**	4.23E+03	2.55E+03	3.13E+03	4.25E+03	2.13E+03	2.44E+05	2.19E+03	1.80E+03
F20	Mean	3.45E+03	4.45E+03	5.15E+03	**3.38E+03**	4.52E+03	2.40E+03	3.66E+03	4.58E+03	4.86E+03
	Std	1.18E+03	6.07E+02	9.08E+02	**5.36E+02**	8.51E+02	4.38E+02	1.05E+03	4.99E+02	5.41E+02
F21	Mean	**5.73E+02**	1.60E+03	8.86E+02	7.44E+02	6.48E+02	8.79E+02	8.89E+02	5.89E+02	2.86E+03
	Std	**4.42E+01**	1.97E+02	2.49E+02	7.67E+01	4.12E+01	7.64E+01	8.56E+01	2.82E+02	5.85E+01
F22	Mean	2.29E+04	2.37E+04	2.74E+04	1.94E+04	**1.52E+04**	1.54E+04	3.15E+04	2.99E+04	2.32E+04
	Std	9.40E+03	1.37E+03	3.24E+03	5.12E+03	**8.36E+03**	1.19E+03	1.62E+03	9.17E+02	1.58E+03
F23	Mean	9.74E+02	2.58E+03	1.25E+03	1.17E+03	1.13E+03	1.13E+03	1.25E+03	**7.78E+02**	3.45E+03
	Std	4.50E+01	3.95E+02	2.59E+02	9.83E+01	9.20E+01	8.49E+01	8.17E+01	**1.25E+02**	7.70E+01
F24	Mean	1.50E+03	4.26E+03	1.96E+03	1.95E+03	1.72E+03	1.90E+03	1.83E+03	**1.11E+03**	4.02E+03
	Std	1.02E+02	7.13E+02	2.69E+02	1.68E+02	1.44E+02	1.39E+02	1.16E+02	**4.62E+01**	8.24E+01
F25	Mean	**8.25E+02**	1.29E+03	1.18E+03	8.90E+02	1.26E+03	1.05E+03	1.30E+03	8.29E+02	4.13E+03
	Std	**4.82E+01**	1.02E+02	1.91E+02	5.85E+01	5.01E+01	6.61E+01	1.01E+02	4.86E+01	1.15E+02
F26	Mean	1.16E+04	2.70E+04	1.40E+04	1.53E+04	1.89E+04	1.49E+04	1.45E+04	**5.69E+03**	1.37E+04
	Std	2.20E+03	2.45E+03	2.67E+03	2.28E+03	3.39E+03	5.54E+03	4.07E+03	**3.41E+02**	1.20E+03
F27	Mean	1.02E+03	1.55E+03	**5.00E+02**	1.41E+03	1.25E+03	1.40E+03	1.40E+03	1.48E+03	3.91E+03
	Std	1.24E+02	3.91E+02	**6.08E−05**	1.62E+02	1.18E+02	2.18E+02	1.98E+02	5.82E+02	1.09E+02
F28	Mean	6.32E+02	1.18E+03	**5.00E+02**	7.32E+02	1.31E+03	8.58E+02	1.17E+03	6.41E+02	4.46E+03
	Std	2.95E+01	2.00E+02	**3.80E−05**	4.40E+01	1.18E+02	4.44E+01	1.46E+02	4.20E+01	2.17E+02
F29	Mean	**3.06E+03**	5.50E+03	6.37E+03	4.30E+03	4.98E+03	4.16E+03	5.65E+03	3.16E+03	6.99E+03
	Std	**5.34E+02**	6.93E+02	1.37E+03	5.74E+02	5.08E+02	5.67E+02	7.37E+02	1.18E+03	6.59E+02
F30	Mean	1.57E+04	3.54E+05	3.50E+07	7.70E+05	1.93E+06	1.57E+05	1.74E+07	**7.50E+03**	5.94E+05
	Std	1.00E+04	2.76E+05	7.69E+07	7.21E+05	1.13E+06	7.19E+04	9.74E+06	**4.72E+03**	2.93E+05

**Table 14:** Wilcoxon rank-sum comparison outcomes for IEHO against competing algorithms over the CEC2017 test suite (30-dimensional).

	EQDEHO	EHO	ICPO	ISO	IGKSO	MGKSO	ICSBO	HICPO
F1	0.000(−)	0.000(−)	0.000(−)	0.000(−)	0.000(−)	0.000(−)	0.947(=)	0.000(−)
F3	0.000(−)	0.000(−)	0.000(−)	0.000(−)	0.000(−)	0.000(−)	0.000(−)	0.000(−)
F4	0.000(−)	0.000(−)	0.017(−)	0.000(−)	0.000(−)	0.000(−)	0.460(=)	0.000(−)
F5	0.000(−)	0.000(−)	0.000(−)	0.000(−)	0.000(−)	0.000(−)	0.000(−)	0.000(−)
F6	0.000(−)	0.000(−)	0.000(−)	0.000(−)	0.000(−)	0.000(−)	0.000(+)	0.000(−)
F7	0.000(−)	0.000(−)	0.000(−)	0.000(−)	0.000(−)	0.000(−)	0.000(−)	0.000(−)
F8	0.000(−)	0.000(−)	0.000(−)	0.000(−)	0.000(−)	0.000(−)	0.000(−)	0.000(−)
F9	0.000(−)	0.000(−)	0.000(−)	0.000(−)	0.000(−)	0.000(−)	0.000(+)	0.000(−)
F10	0.026(−)	0.000(−)	0.001(−)	0.000(−)	0.000(+)	0.574(=)	0.663(=)	0.578(=)
F11	0.000(−)	0.000(−)	0.000(+)	0.000(−)	0.000(−)	0.000(−)	0.000(+)	0.000(−)
F12	0.000(−)	0.000(−)	0.000(−)	0.000(−)	0.000(−)	0.000(−)	0.002(−)	0.000(−)
F13	0.000(−)	0.000(−)	0.000(−)	0.000(−)	0.000(−)	0.000(−)	0.007(−)	0.000(−)
F14	0.000(−)	0.000(−)	0.000(−)	0.000(−)	0.000(−)	0.000(−)	0.000(+)	0.000(−)
F15	0.000(−)	0.000(−)	0.000(−)	0.000(−)	0.000(−)	0.000(−)	0.000(+)	0.000(−)
F16	0.000(−)	0.000(−)	0.001(−)	0.000(−)	0.001(−)	0.000(−)	0.000(−)	0.000(−)
F17	0.000(−)	0.000(−)	0.000(−)	0.000(−)	0.133(=)	0.000(−)	0.000(−)	0.000(−)
F18	0.000(−)	0.000(−)	0.000(−)	0.000(−)	0.000(−)	0.000(−)	0.000(−)	0.000(−)
F19	0.000(−)	0.000(−)	0.000(−)	0.000(−)	0.000(−)	0.000(−)	0.000(+)	0.000(−)
F20	0.000(−)	0.000(−)	0.000(−)	0.000(−)	0.000(−)	0.000(−)	0.000(−)	0.000(−)
F21	0.000(−)	0.000(−)	0.000(−)	0.000(−)	0.000(−)	0.000(−)	0.000(−)	0.000(−)
F22	0.000(−)	0.000(−)	0.040(−)	0.000(−)	0.112(=)	0.000(−)	0.597(=)	0.000(−)
F23	0.000(−)	0.000(−)	0.000(−)	0.000(−)	0.000(−)	0.000(−)	0.917(=)	0.000(−)
F24	0.000(−)	0.000(−)	0.000(−)	0.000(−)	0.000(−)	0.000(−)	0.000(−)	0.000(−)
F25	0.000(−)	0.053(=)	0.296(=)	0.000(−)	0.000(−)	0.000(−)	0.051(=)	0.000(−)
F26	0.000(−)	0.000(−)	0.001(−)	0.251(=)	0.009(−)	0.000(−)	0.000(+)	0.000(−)
F27	0.000(−)	0.000(−)	0.000(−)	0.000(+)	0.000(−)	0.000(−)	0.000(+)	0.000(−)
F28	0.000(−)	0.000(−)	0.006(=)	0.000(−)	0.000(−)	0.000(−)	0.072(=)	0.000(−)
F29	0.000(−)	0.000(−)	0.000(+)	0.000(−)	0.000(−)	0.000(−)	0.807(=)	0.000(−)
F30	0.000(−)	0.579(=)	0.000(−)	0.000(−)	0.000(−)	0.000(−)	0.008(+)	0.000(−)
+/=/−	0/1/28	0/2/27	2/2/25	1/1/27	1/2/27	0/1/28	9/8/12	0/1/28

**Table 15:** Wilcoxon rank-sum comparison outcomes for IEHO against competing algorithms over the CEC2017 test suite (50-dimensional).

	EQDEHO	EHO	ICPO	ISO	IGKSO	MGKSO	ICSBO	HICPO
F1	0.000(+)	0.000(+)	0.000(+)	0.000(−)	0.589(=)	0.000(−)	0.000(+)	0.000(−)
F3	0.000(−)	0.000(−)	0.000(+)	0.000(−)	0.371(=)	0.000(−)	0.000(+)	0.000(−)
F4	0.000(−)	0.000(−)	0.000(−)	0.000(−)	0.000(−)	0.000(−)	0.060(=)	0.000(−)
F5	0.000(−)	0.000(−)	0.000(−)	0.000(−)	0.000(−)	0.000(−)	0.107(=)	0.000(−)
F6	0.000(−)	0.000(−)	0.000(−)	0.000(−)	0.000(−)	0.000(−)	0.000(+)	0.000(−)
F7	0.000(−)	0.000(−)	0.000(−)	0.000(−)	0.000(−)	0.000(−)	0.000(−)	0.000(−)
F8	0.000(−)	0.000(−)	0.000(−)	0.000(−)	0.000(−)	0.000(−)	0.002(−)	0.000(−)
F9	0.000(−)	0.000(−)	0.000(−)	0.000(−)	0.000(−)	0.000(−)	0.000(+)	0.000(−)
F10	0.012(+)	0.761(=)	0.000(+)	0.000(+)	0.000(+)	0.162(=)	0.501(=)	0.020(+)
F11	0.124(=)	0.000(−)	0.084(=)	0.000(−)	0.056(=)	0.000(−)	0.000(+)	0.000(−)
F12	0.000(−)	0.000(−)	0.000(−)	0.000(−)	0.000(−)	0.000(−)	0.000(−)	0.000(−)
F13	0.000(−)	0.000(−)	0.000(−)	0.000(−)	0.006(−)	0.000(−)	0.255(=)	0.000(−)
F14	0.000(−)	0.000(−)	0.000(−)	0.000(−)	0.000(−)	0.000(−)	0.000(+)	0.000(−)
F15	0.000(−)	0.000(−)	0.012(−)	0.000(−)	0.000(−)	0.000(−)	0.000(+)	0.000(−)
F16	0.000(−)	0.000(−)	0.000(−)	0.000(−)	0.000(−)	0.000(−)	0.000(−)	0.000(−)
F17	0.000(−)	0.000(−)	0.224(=)	0.091(=)	0.000(−)	0.000(−)	0.000(−)	0.000(−)
F18	0.000(−)	0.000(−)	0.000(−)	0.000(−)	0.000(−)	0.000(−)	0.006(−)	0.000(−)
F19	0.000(−)	0.000(−)	0.000(−)	0.000(−)	0.000(−)	0.000(−)	0.009(+)	0.000(−)
F20	0.000(−)	0.000(−)	0.418(=)	0.000(−)	0.600(=)	0.000(−)	0.000(−)	0.000(−)
F21	0.000(−)	0.000(−)	0.000(−)	0.000(−)	0.000(−)	0.000(−)	0.007(−)	0.000(−)
F22	0.000(−)	0.000(−)	0.000(−)	0.173(=)	0.000(−)	0.000(−)	0.006(−)	0.000(−)
F23	0.000(−)	0.000(−)	0.000(−)	0.000(−)	0.000(−)	0.000(−)	0.000(−)	0.000(−)
F24	0.000(−)	0.000(−)	0.000(−)	0.000(−)	0.000(−)	0.000(−)	0.000(−)	0.000(−)
F25	0.000(−)	0.235(=)	0.891(=)	0.000(−)	0.000(−)	0.000(−)	0.058(=)	0.000(−)
F26	0.000(−)	0.000(−)	0.000(−)	0.000(−)	0.129(=)	0.000(−)	0.000(+)	0.000(−)
F27	0.000(−)	0.000(+)	0.000(−)	0.000(−)	0.000(−)	0.000(−)	0.000(+)	0.000(−)
F28	0.000(−)	0.000(+)	0.016(−)	0.000(−)	0.000(−)	0.000(−)	0.078(=)	0.000(−)
F29	0.000(−)	0.000(−)	0.000(−)	0.000(−)	0.000(−)	0.000(−)	0.081(=)	0.000(−)
F30	0.000(−)	0.000(−)	0.000(−)	0.000(−)	0.000(−)	0.000(−)	0.549(=)	0.000(−)
+/=/−	2/1/26	3/2/24	3/4/22	1/2/26	1/5/23	0/1/28	10/8/11	1/0/28

**Table 16:** Wilcoxon rank-sum comparison outcomes for IEHO against competing algorithms over the CEC2017 test suite (100-dimensional).

	EQDEHO	EHO	ICPO	ISO	IGKSO	MGKSO	ICSBO	HICPO
F1	0.000(−)	0.000(−)	0.165(=)	0.137(=)	0.000(−)	0.000(−)	0.000(+)	0.000(−)
F3	0.000(+)	0.008(−)	0.000(+)	0.000(−)	0.000(+)	0.000(−)	0.000(+)	0.000(−)
F4	0.000(−)	0.000(−)	0.000(−)	0.015(−)	0.000(−)	0.000(−)	0.258(=)	0.000(−)
F5	0.000(−)	0.000(−)	0.000(−)	0.000(−)	0.000(−)	0.000(−)	0.000(+)	0.000(−)
F6	0.000(−)	0.000(−)	0.000(−)	0.000(−)	0.000(−)	0.000(−)	0.000(+)	0.000(−)
F7	0.000(−)	0.000(−)	0.000(−)	0.000(−)	0.000(−)	0.000(−)	0.000(−)	0.000(−)
F8	0.000(−)	0.000(−)	0.000(−)	0.000(−)	0.000(−)	0.000(−)	0.000(−)	0.000(−)
F9	0.000(−)	0.000(−)	0.000(−)	0.000(−)	0.000(−)	0.000(−)	0.000(+)	0.000(−)
F10	0.906(=)	0.317(−)	0.535(=)	0.000(+)	0.000(+)	0.000(−)	0.231(=)	0.027(+)
F11	0.000(−)	0.000(−)	0.000(−)	0.000(−)	0.000(−)	0.000(−)	0.000(+)	0.000(−)
F12	0.000(−)	0.000(−)	0.000(−)	0.000(−)	0.000(−)	0.000(−)	0.021(−)	0.000(−)
F13	0.000(−)	0.000(−)	0.000(−)	0.000(−)	0.035(−)	0.000(−)	0.352(=)	0.000(−)
F14	0.000(−)	0.000(−)	0.000(−)	0.000(−)	0.000(−)	0.000(−)	0.051(=)	0.000(−)
F15	0.000(−)	0.000(−)	0.000(−)	0.000(−)	0.008(−)	0.000(−)	0.882(=)	0.000(−)
F16	0.000(−)	0.000(−)	0.000(−)	0.000(−)	0.000(−)	0.000(−)	0.014(−)	0.000(−)
F17	0.000(−)	0.000(−)	0.000(−)	0.000(−)	0.000(−)	0.000(−)	0.000(−)	0.000(−)
F18	0.000(−)	0.000(−)	0.000(−)	0.000(−)	0.000(−)	0.000(−)	0.015(−)	0.000(−)
F19	0.000(−)	0.001(−)	0.000(−)	0.615(=)	0.066(=)	0.000(−)	0.627(=)	0.000(−)
F20	0.000(−)	0.020(−)	0.641(=)	0.001(−)	0.000(+)	0.294(=)	0.003(−)	0.000(−)
F21	0.000(−)	0.000(−)	0.000(−)	0.000(−)	0.000(−)	0.000(−)	0.000(−)	0.000(−)
F22	0.784(=)	0.004(−)	0.000(+)	0.003(+)	0.000(+)	0.000(−)	0.013(−)	0.054(=)
F23	0.000(−)	0.000(−)	0.000(−)	0.000(−)	0.000(−)	0.000(−)	0.000(+)	0.000(−)
F24	0.000(−)	0.000(−)	0.000(−)	0.000(−)	0.000(−)	0.000(−)	0.000(+)	0.000(−)
F25	0.000(−)	0.000(−)	0.000(−)	0.000(−)	0.000(−)	0.000(−)	0.836(=)	0.000(−)
F26	0.000(−)	0.000(−)	0.000(−)	0.000(−)	0.000(−)	0.000(−)	0.000(+)	0.000(−)
F27	0.000(−)	0.000(+)	0.000(−)	0.000(−)	0.000(−)	0.000(−)	0.000(−)	0.000(−)
F28	0.000(−)	0.000(+)	0.000(−)	0.000(−)	0.000(−)	0.000(−)	0.900(=)	0.000(−)
F29	0.000(−)	0.000(−)	0.000(−)	0.000(−)	0.000(−)	0.000(−)	0.918(=)	0.000(−)
F30	0.000(−)	0.000(−)	0.000(−)	0.000(−)	0.000(−)	0.000(−)	0.000(+)	0.000(−)
+/=/−	1/2/26	2/1/26	2/3/24	2/2/25	4/2/23	0/1/28	9/9/11	1/1/27

**Table 17:** Friedman test for each algorithm.

		IEHO	EQDEHO	EHO	ICPO	ISO	IGKSO	MGKSO	ICSBO	HICPO
**D = 30**	Avg.rank	1.76	6.66	7.2	3.00	5.14	4.72	6.31	2.52	7.69
	sort	1	7	8	3	5	4	6	2	9
**D = 50**	Avg.rank	1.93	6.65	6.75	3.37	5.31	4.27	6.83	2.68	7.34
	sort	1	6	7	3	5	4	8	2	9
**D = 100**	Avg.rank	2.24	6.83	6.72	4.10	5.69	4.28	7.52	2.59	5.03
	sort	1	8	7	3	6	4	9	2	5

**Table 18:** Time cost per algorithm for 500-generation runs (s).

	EHO	IEHO	EQDEHO	ICPO	ISO	IGKSO	MGKSO	ICSBO	HICPO
F1	280.32	447.28	417.07	**87.78**	228.64	476.41	1222.1	555.53	91.72
F3	232.62	445.39	399.1	**83.88**	224.63	497.42	529.45	513.4	131.74
F4	265.75	395.67	393.25	**85**	244.29	400.48	579.34	489.71	102.71
F5	296.23	457.86	413.2	**96.46**	269.96	481.2	610.72	519.84	121.67
F6	404.05	486.8	582.14	**132.19**	391.53	660.14	821.29	581.94	141.25
F7	319.98	428.48	437.44	196.67	260.55	489.37	687.23	559.85	**107.3**
F8	287.25	431.31	425.86	200.77	244.95	487.08	676.27	547.72	**108.35**
F9	277.45	419.81	453.21	**96.16**	257.5	518.99	632.29	533.08	118.16
F10	325.88	477.51	450.03	113.15	267.157	463.66	566.3	534.7	**108.49**
F11	302.34	440.78	414.15	**89.9**	237.77	448.6	549.07	518.51	148.11
F12	255.35	461.19	448.57	**96.27**	262.55	489.87	537.14	538.96	204.882
F13	328.4	412.33	450.77	**89.11**	243.68	564.88	557.17	545.98	389.227
F14	321.64	456.45	480.78	**123.49**	289.8	512.8	601.51	565.64	175.84
F15	289.2	434.59	449.88	**89.01**	225.98	473.99	577.02	533.62	130.88
F16	279.87	430.71	427.15	**98.06**	261.21	463.75	512.49	584.6	158.12
F17	368.44	482.69	541.21	**131.69**	336.8	575.7	575.49	567.61	166.12
F18	299.27	440.84	1424.96	**87.38**	244.73	521.49	514.63	575.48	148.81
F19	909.37	693.5	1115.35	**284.26**	740.9	1079.8	1045.73	889.08	355.13
F20	405.84	482.46	554.03	**142.69**	338.55	677.62	628.57	598.49	211.25
F21	430.93	580.92	614.23	**132.36**	401.32	654.9	703.53	658.95	216.4
F22	443.34	506.62	638.39	**143.41**	432.65	663.24	715.88	696.18	225.29
F23	507.69	533.71	697.24	**155.39**	446.45	740.25	823.85	715.11	238.78
F24	612.84	538.02	703.47	**167.83**	519.36	703.23	845.85	735.58	230.7
F25	539.43	538.02	710.37	**154.83**	478.08	755.01	726.08	694.79	243.26
F26	638.8	624.1	819.08	**162.44**	552.49	857.8	871.02	791.78	292.1
F27	706	634.56	942.44	**198.02**	618.47	875.86	1009.8	826.67	228.8
F28	637.59	556.8	809.78	**151.69**	562.92	829.71	871.35	751.39	280.47
F29	501.16	525.58	697.48	**155.01**	444.11	724.1	789.44	678.13	229.3
F30	1048.31	725.52	1241.12	**338.25**	931.4	1228.77	1121.23	994.69	385.85
	0	0	0	**26**	0	0	0	0	3

**Table 19:** Time to reach equivalent accuracy across algorithms (s).

	EHO	IEHO	EQDEHO	ICPO	ISO	IGKSO	MGKSO	ICSBO	HICPO
F1	324.45	**100.40**	185.83	187.48	2510.91	765.48	4826.30	106.71	1419.99
F3	291.95	**9.46**	26.20	51.51	55.62	7.32	28.67	10.57	9.07
F4	313.61	730.31	77.23	828.92	2766.27	3264.44	3840.27	**46.04**	1403.27
F5	370.77	16.19	**2.89**	307.22	22.64	7.07	73.29	7.38	264.91
F6	510.28	**11.52**	522.34	73.13	151.48	138.90	3523.85	15.17	221.15
F7	344.47	**12.36**	435.05	25.42	151.42	38.71	643.91	12.49	659.08
F8	366.87	467.41	2080.12	1797.57	97.17	555.37	219.39	**82.49**	1622.98
F9	328.84	**4.68**	772.67	17.80	155.82	9.73	57.57	7.97	65.84
F10	422.64	0.50	0.79	0.15	22.97	0.98	2.07	0.87	**0.20**
F11	286.91	300.95	3362.70	**64.46**	135.44	484.04	649.38	290.05	1654.98
F12	382.31	**155.32**	486.22	1741.68	1053.60	5254.22	3683.10	162.86	1697.31
F13	322.13	35.58	26.04	**17.59**	115.66	17.81	737.89	19.71	268.30
F14	453.70	**5.87**	8.33	12.70	158.41	12.89	210.08	6.90	1820.40
F15	346.37	**62.08**	1132.83	1587.82	138.42	4741.82	732.48	71.27	1718.29
F16	353.59	81.17	6.41	**5.40**	138.42	5.50	43.82	70.62	526.37
F17	469.88	**4.16**	10.37	43.86	11.52	30.33	32.39	7.70	63.23
F18	354.13	9.11	16.34	**7.13**	131.01	119.92	1547.38	11.22	1888.79
F19	1184.76	19.31	**10.18**	36.06	341.28	21.34	1289.69	30.37	4927.45
F20	516.58	208.34	23.64	27.30	26.91	**13.17**	65.89	15.43	199.60
F21	612.04	196.06	591.65	49.74	**13.67**	23.51	149.74	580.39	1443.03
F22	620.23	**9.40**	10.87	10.51	20.45	10.49	10.50	11.42	20.26
F23	717.70	266.39	**172.60**	2532.10	6203.10	2846.27	3882.09	2905.95	2671.41
F24	814.37	41.49	2457.03	43.40	17.45	**5.99**	31.24	61.59	1407.13
F25	779.92	13,265.41	1957.79	5103.23	1801.37	2953.83	815.38	**114.79**	2549.20
F26	853.06	**11.50**	780.38	16.65	6759.40	115.06	963.87	335.70	18.87
F27	962.26	**102.46**	883.02	3838.57	8034.50	4152.00	440.01	323.17	3218.97
F28	884.72	62.37	134.49	39.44	167.22	34.64	281.00	**27.94**	1477.96
F29	746.70	**35.07**	1183.93	65.96	43.78	77.70	1004.14	91.71	301.28
F30	1419.00	1699.46	4043.26	12,281.54	3837.60	1432.27	1601.28	**1311.77**	5429.75
	0	13	3	4	1	2	0	**5**	1

## Data Availability

The original contributions presented in this study are included in the article. Further inquiries can be directed to the corresponding author.
